# Subtypes in addiction and their neurobehavioral profiles across three functional domains

**DOI:** 10.1038/s41398-023-02426-1

**Published:** 2023-04-18

**Authors:** Gunner Drossel, Leyla R. Brucar, Eric Rawls, Timothy J. Hendrickson, Anna Zilverstand

**Affiliations:** 1 Graduate Program in Neuroscience, University of Minnesota, Minneapolis, MN USA; 2 Department of Psychiatry and Behavioral Sciences, University of Minnesota, Minneapolis, MN USA; 3 University of Minnesota Informatics Institute, University of Minnesota, Minneapolis, MN USA; 4 Masonic Institute for the Developing Brain, University of Minnesota, Minneapolis, MN USA; 5 Medical Discovery Team on Addiction, University of Minnesota, Minneapolis, MN USA

**Keywords:** Diagnostic markers, Addiction

## Abstract

Rates of return to use in addiction treatment remain high. We argue that the development of improved treatment options will require advanced understanding of individual heterogeneity in Substance Use Disorders (SUDs). We hypothesized that considerable individual differences exist in the three functional domains underlying addiction—approach-related behavior, executive function, and negative emotionality. We included *N* = 593 participants from the enhanced Nathan Kline Institute-Rockland Sample community sample (ages 18–59, 67% female) that included *N* = 420 Controls and *N* = 173 with past SUDs [54% female*; N* = *75 Alcohol Use Disorder (AUD) only*, *N* = *30 Cannabis Use Disorder (CUD) only*, and *N* = *68 Multiple SUDs*]. To test our a priori hypothesis that distinct neuro-behavioral subtypes exist within individuals with past SUDs, we conducted a latent profile analysis with all available phenotypic data as input (74 subscales from 18 measures), and then characterized resting-state brain function for each discovered subtype. Three subtypes with distinct neurobehavioral profiles were recovered (*p* < 0.05, Cohen’s *D*: 0.4–2.8): a “Reward type” with higher approach-related behavior (*N* = 69); a “Cognitive type” with lower executive function (*N* = 70); and a “Relief type” with high negative emotionality (*N* = 34). For those in the Reward type, substance use mapped onto resting-state connectivity in the Value/Reward, Ventral-Frontoparietal and Salience networks; for the Cognitive type in the Auditory, Parietal Association, Frontoparietal and Salience networks; and for the Relief type in the Parietal Association, Higher Visual and Salience networks (*p*_FDR_ < 0.05). Subtypes were equally distributed amongst individuals with different primary SUDs (*χ*^2^ = 4.71, *p* = 0.32) and gender (*χ*^2^ = 3.44, *p* = 0.18). Results support functionally derived subtypes, demonstrating considerable individual heterogeneity in the multi-dimensional impairments in addiction. This confirms the need for mechanism-based subtyping to inform the development of personalized addiction medicine approaches.

## Introduction

In the United States, substance use disorders (SUDs) are a major source of morbidity and mortality. The lifetime prevalence of SUDs has been estimated to be as high as 30% [1]. Currently, approximately 40.3 million people in the U.S. have a SUD diagnosis [2]. Independent of their primary substance of choice, over two-thirds of individuals return to use within weeks to months of initiating treatment, and up to 85% of individuals return to substance use within one year of treatment completion [2–4]. Research suggests that this is due to suboptimal treatment options, low confidence in their effectiveness, and restricted access to treatment facilities [5, 6]. We argue that alleviating this urgent situation; specifically, improving the effectiveness of existing treatments, will require a better understanding of the mechanisms underlying the vulnerability for SUDs to develop and to persist. Furthermore, we recognize that the underlying neurobehavioral mechanisms are likely to be heterogeneous across individuals with SUDs, and understanding this heterogeneity will be critical for conceptualizing advances in treatment.

Prominent addiction theories have stressed the importance of three neurobehavioral mechanisms of persistence in addiction: (1) altered incentive salience (also reward- or approach-related behavior), (2) lower executive function, and (3) increased negative emotionality [7, 8]. Recent evidence from individuals with alcohol use disorder (AUD) has empirically demonstrated the validity of this three-mechanism model of addiction [9, 10]. It has further been argued that these three mechanisms are dependent on each other, going back to studies showing that substances of abuse impair all of the neurobiological systems underlying them [11–14]. However, contrary to this assumption, decades-old research from psychology provided evidence for the functional independence of the approach-related behavior, executive function and negative emotionality domains [15, 16]. Accordingly, we propose to investigate if function on these three domains is at least partially independent, such that different combinations of impairments on these domains may underlie addiction persistence.

Efforts to investigate heterogeneity and identify subtypes within populations with SUDs have largely focused on clinical/diagnostic data that assessed addiction severity (and sometimes psychiatric comorbidity), rather than the underlying mechanisms [17–23]. Through literature searches (PubMed, Google Scholar), we have identified over 120 published studies that have subtyped individuals with SUDs using symptom scales (e.g., AUDIT, ASI, AUDADIS-IV, SSADDA, DSM-IV, ICD-10^1^). These initial efforts to define subgroups or “subtypes” in individuals with SUDs consistently found that substance users can be separated into a milder “late onset” type, consisting of individuals characterized by low psychiatric comorbidity versus a more severe “early onset” type with high psychiatric comorbidity, more poly-substance use, and worse treatment prognosis [24–33]. In contrast, previous research characterizing individual differences in the multi-dimensional mechanisms (rather than symptom presentation) of addiction is extremely limited. Only two studies have investigated heterogeneity in impulsivity and found “impulsive” versus “non-impulsive” types [34, 35], while two other studies differentiated individuals based on drinking motives and found a “reward” (pleasure seeking) versus “relief” (drinking to cope) motivational type [36, 37]. Thus, even within the space of mechanism-based subtyping, existing literature is limited to a narrowly-focused space of the mechanisms underlying addiction, rather than applying a broad, multi-dimensional approach.

The goal of the current study was to use a multi-dimensional “mechanism-based” subtyping approach, using data informing the three functional domains implicated in addiction persistence: (1) approach-related behavior, (2) executive function, and (3) negative emotionality [7, 8]. We leveraged the enhanced Nathan Kline Institute-Rockland Sample (NKI-RS), a dataset which includes a comprehensive phenotypic assessment that broadly characterized individual function on these three domains, provided a clinical characterization of the sample, and included neuroimaging data. This large, heterogeneous community sample included participants with varying past SUD diagnoses. We hypothesized considerable individual heterogeneity in the impairments on the three functional domains of interest. We further hypothesized that individual heterogeneity underlying addiction would have a large trait-component. We hence expected to find the existence of at least three separable neuro-behaviorally distinct subtypes in past substance users: a “reward type” with higher reward-seeking, a “cognitive type” with impaired executive function, and a “relief type” defined by the use of substance to cope with high negative affect. Finally, we expected that each discovered subtype would be characterized by corresponding, unique neurobiological impairments of resting-state brain function.

## Participants and methods

### Participants

We analyzed data from the NKI-RS study that was collected at the NKI in New York, from 2012 to 2016 [38]. All study procedures and informed consent forms, including consent to share de-identified data, were approved by the Nathan Kline Institute Institutional Review Board (IRB) in accordance with the Declaration of Helsinki. After removing measures with >10% missing data, *N* = 612 participants ages 18–59 had complete phenotypic data. Of these participants, *N* = 420 were Controls (participants with neither a past nor a current SUD diagnosis), *N* = 19 had current SUDs, and *N* = 173 were participants with past SUDs (54% females). Due to their low number, individuals with current SUDs were excluded, leaving *N* = 593 participants (67% female) in the sample. Within individuals with past SUDs, *N* = 75 were diagnosed with past AUD only, *N* = 30 were diagnosed with past Cannabis Use Disorder (CUD) only and *N* = 68 were diagnosed with Multiple SUD diagnoses, see Supplementary Table 1. Detailed demographic information is provided in Table 1a. All clinical diagnoses (SUD and comorbid diagnoses) were assessed with the Structured Clinical Interview for DSM-IV [39]. Tobacco Use Disorder was not assessed, due to the low predictive validity of this diagnosis [40].

**Table 1:** (a) Demographics and diagnoses by past SUD; (b) clinical characteristics by past SUD.

	Past SUD (any) (*N* = 173)	Past AUD (*N* = 75)	Past CUD (*N* = 30)	Past multi-UD (MUD) (*N* = 68)	Controls (*N* = 420)
(a)					
Gender: M/F	78/94***	24/51	15/15**	39/29***	115/305
Age (years)	40.1 (13)	40.4 (12.7)	34.4 (13.1)	42.3 (12.7)*	38.0 (13.3)
Race (White/African American/Other)	108/46/19**	51/17/7	16/10/4*	41/18/9 *	316/68/36
Education—years completed	14.7 (2.1)***	14.6 (2)	13.8 (2)***	14.6 (2.2)**	15.4 (2.1)
Current smoker (# participants)	33***	11*	8***	14***	28
Tobacco (including smokeless) use—times using/day	3.3 (12.6)***	3.8 (18.1)**	2.9 (5.0)**	3.0 (6.1)***	0.9 (3.3)
Past 6 months days drunk (# days)	4.1 (8.7)*	4.4 (7.0)*	2.1 (3.1)	4.7 (11.6)*	2.5 (7.4)
Past 6 months nonmedicinal substance use (# days)	8.8 (27.0)***	2.5 (6.7)	20.8 (45.9)***	10.5 (28.2)***	2.7 (15.4)
# Past internalizing disorders (%)	0.36 (0.61)*	0.39 (0.61)	0.5 (0.73)*	0.23 (0.54)	0.25 (0.53)
Anxiety (*N*)	0	0	0	0	1
Phobia (*N*)	3	0	2	1	4
Panic disorder (*N*)	5	2	1	2	10
Obsessive compulsive disorder (*N*)	1	1	0	0	4
Depression or dysthymic disorder (*N*)	45	21	9	15	72
Eating disorder or body dysmorphia (*N*)	4	3	1	0	4
Posttraumatic stress disorder (*N*)	3	2	1	0	9
# Past externalizing disorders (%)	0.05 (0.21)	0.01 (0.12)	0.07 (0.25)	0.07 (0.26)**	0.02 (0.14)
Attention-deficit/hyperactivity disorder (*N*)	9	1	2	6	6
Bipolar disorder (*N*)	2	0	0	2	0
Other psychosis (e.g., schizophrenia) (*N*)	0	0	0	0	0
# Current internalizing disorders (%)	0.23 (0.56)	0.25 (0.64)	0.023 (0.50)	0.21 (0.51)	0.15 (0.47)
Anxiety (*N*)	15	5	4	6	15
Phobia (*N*)	6	3	1	2	14
Panic disorder (*N*)	3	1	2	0	3
Obsessive compulsive disorder (*N*)	3	1	0	2	4
Depression or dysthymic disorder (*N*)	12	7	1	5	20
Eating disorder or body dysmorphia (*N*)	3	2	0	1	4
Posttraumatic stress disorder (*N*)	6	3	0	3	6
# Current externalizing disorders (%)	0.04 (0.20)*	0.01 (0.12)	0.07 (0.25)*	0.06 (0.24)	0.03 (0.17)
Attention-deficit/hyperactivity disorder (*N*)	7	1	2	4	11
Bipolar disorder (*N*)	1	0	1	0	0
Other psychosis (e.g., schizophrenia) (*N*)	0	0	0	0	2

(b)					
ASR: Anxious/Depressed (past 6 months)	55.2 (7.7)**	55.5 (7.7)*	55.8 (9.0)	54.7 (7.1)	53.5 (6.8)
ASR: Withdrawn (past 6 months)	54.4 (6.5)	54.2 (6.9)	56.0 (7.3)*	53.8 (5.7)	53.4 (6.3)
ASR: Somatic Complaints (past 6 months)	55.2 (6.0)**	54.3 (5.3)	55.1 (5.9)	56.3 (6.7)***	53.5 (6.0)
ASR: Thought Problems (past 6 months)	53.0 (5.1)*	53.1 (5.1)	54.1 (5.9)*	52.5 (4.8)	52.0 (5.0)
ASR: Attention Problems (past 6 months)	55.6 (7.0)*	56.2 (7.3)*	55.4 (6.4)	55.0 (7.1)	54.1 (6.7)
ASR: Aggressive Behavior (past 6 months)	53.9 (5.4)**	53.8 (4.8)*	53.0 (5.1)	54.5 (6.0)**	52.6 (5.3)
ASR: Rule-Breaking Behavior (past 6 months)	55.8 (6.4)***	55.7 (6.6)***	56.3 (6.4)**	55.7 (6.4)**	53.1 (5.7)
ASR: Intrusive (past 6 months)	53.0 (4.3)	53.1 (4.4)	51.8 (3.4)	53.3 (4.6)	52.4 (4.9)
Beck Depression Inventory Total (past 2 weeks)	7.8 (7.6)***	8.1 (7.6)**	8.2 (9.2)	7.3 (6.9)	5.5 (6.7)
Trauma Symptom Checklist (past 2 months)	24.6 (15.4)***	24.5 (15.3)***	23.6 (15.5)*	25.0 (15.8)***	18.5 (13.3)
CAARS: Inattention/Memory Problems	48.3 (9.8)	48.5 (9.8)	47.7 (10.1)	48.3 (9.7)	47.2 (8.9)
CAARS: Hyperactivity/Restlessness	48.6 (8.7)***	48.4 (9.5)**	**47.1 (8.9)**	49.4 (7.6)***	45.8 (7.6)
CAARS: Impulsivity/Emotional Liability	48.2 (7.7)***	48.3 (7.4)***	47.7 (9.0)*	48.2 (7.4)***	45.0 (7.0)
CAARS: Problems with Self-Concept	49.0 (9.9)***	49.6 (10.2)**	48.4 (11.5)	48.6 (8.8)*	46.1 (8.8)
CAARS: ADHD Index	48.3 (9.6)***	48.3 (9.6)**	47.4 (11.0)	48.8 (9.2)***	45.0 (8.1)

*SUD* substance use disorder, *AUD* alcohol use disorder, *CUD* cannabis use disorder, *MUD* multi use disorders, *ASR* Adult Self Report, *CAARS* Conners’ Adult ADHD Rating Scales.

For Table (a), MUD included *N* = 60 alcohol, 6 amphetamine, 54 cannabis, 27 cocaine, 5 hallucinogenic, 1 inhalant, 3 opioid, 1 phencyclidine, 2 sedative/hypnotic, 1 polysubstance undefined.

Mean (SD); bold font with asterisk denotes that Use Disorder group differs from Controls; *p < 0.05, **p < 0.01, ***p < 0.001.

For Table (b), scores from clinical assessments.

Independent samples *t* test was used to compare each Use Disorder group to Controls.

Columns 2–4: bold font with asterisk denotes significant difference to Controls; *p* < 0.05*, *p* < 0.01**, *p* < 0.001***.

### Phenotypic measures

The NKI-RS data set phenotyped participants using self-report measures and tasks that assessed behavior, affect, clinical symptoms and cognition. We included all available 74 subscales (derived from 18 different measures) that had <10% missing data (listed in Supplementary Table 2). We did therefore not perform any a priori selection of measures based on theoretical considerations, but rather intended to model the entire phenotypic space.

### Factor analysis of phenotypic data

To reduce the measured phenotypic space to a set of latent constructs, an exploratory factor analysis (EFA) was conducted that included all study participants (*N* = 593) to model the full range of phenotypic variance. This analysis was very well powered (*N* > 500; participant/variable ratio=8.0), based on sample size recommendations for EFA [41]. We used Monte Carlo permutation analysis (parallel analysis) [42] to determine the number of factors statistically significant at *p* < 0.05 [43]. Factors were extracted using maximum likelihood as calculated by the expectation-maximization algorithm. Additionally, an oblimin rotation was used to allow for correlated factors. The use of oblimin rotation is critical to data reduction over a large phenotypic variable space, as many factors are expected to be separable but closely related. EFA was conducted in R using the “psych” package [44]. Details on why this method was chosen over other data reduction schemes are included in the Supplementary Methods. One participant (*N* = 1) was deemed an outlier (>3 SD from mean) on their factor loading for “*general psychiatric symptoms*” and was removed from further analysis.

### Extraction of functional domains

To visualize how latent constructs (factors generated by the EFA) were related to each other, we used a spring-embedded plot. Each factor was visualized as a node. Edges connecting nodes act as “springs” between pairs of nodes. Nodes (factors) that were more strongly correlated were plotted closer together in two-dimensional space, to provide an intuitive visual representation of the factor correlations [45]. The algorithm was run on the full correlation matrix from the EFA, using MATLAB’s “graph” object. Only minimal correlation weights were used, such that each factor was connected to at least one other factor.

### Subtyping analysis in past SUDs

To recover distinct subgroups within individuals with past SUD diagnosis (*N* = 173), subtypes were determined using latent profile analysis (LPA) on the EFA latent factors scores. LPA, a form of Gaussian-mixture modeling, builds a model of the data to find subtypes within the modeled multi-dimensional data variable space [46], which here is the variable space defined by the EFA. LPA was performed in R (4.0.5) using the “mclust” package (version 5.7.4) [47, 48]. The model was initialized by model-based hierarchical clustering, and an expectation-maximization algorithm was used to fit the model by assigning posterior probabilities to variable distributions [49, 50]. Bayesian Information Criteria (BIC) were computed for each parameterized model given the log-likelihood, the dimension of data, and the number of mixture components in the model. The model with the lowest BIC and non-negative BIC difference was selected [51]. The bootstrap likelihood ratio test (BLRT) was also used to evaluate model fit to models with *k*−1 profiles [52]. The power for this analysis (*N* = 173; 12 input variables that are continuous; *N* = 34 in the smallest observed class; observed effect size for profile differentiation: *d* = 1.7–2.8) was excellent (close to 1) according to simulations [51, 53].

### Phenotypic characterization of recovered subtypes

To characterize the recovered subtypes with regards to their phenotypic profiles, we tested for statistically significant differences between the recovered types on the extracted EFA factors using independent samples *t* test with Holm–Bonferroni correction. We also correlated individual EFA factor scores with current non-medicinal substance use (number of days in the past 6 months) and binge-drinking (number of days in the past 6 months; men: 5 or more drinks per day, women: 4 or more drinks per day) with Holm-Bonferroni correction.

### Resting-state functional connectivity per subtype

To characterize resting-state brain function underlying return to substance use and to investigate if the neural correlates of return to use would differ between subtypes, we conducted a resting-state functional connectivity analysis of the neural correlates of continued substance use for each subtype separately. We included the subset of participants with complete NIDA Quick Screen and Adult Self Report (ASR) data and more than 5 min of resting-state data after denoising (*Past SUD N* = *104, 59% Female; Controls N* = *302, 74% Female*). We used the matchControls function from the R “e1071” library [54] to determine a separate age- and gender-matched control group for each subtype. We also assessed urine screens on MRI day (Supplementary Table 3).

Imaging data was acquired on a Siemens Trio 3.0 T scanner (Siemens Healthcare GmbH, Erlangen, Germany) equipped with a 32-channel head coil [38]. For each participant, a T1-weighted (T1w) magnetization prepared gradient echo sequence was acquired for a structural image (repetition time=1900 ms, echo time=2.52 ms, flip angle=9°, 176 slices, 1 mm^3^ isotropic voxels). To analyze resting-state functional connectivity, we used the “REST645” scan that was acquired using a multiband echo-planar imaging sequence with the following parameters: multiband factor=4, volumes=900, repetition time=645 ms, echo time=30 ms, flip angle=60°, 3 mm^3^ isotropic voxels.

For data quality control, MRI Quality Control tool (MRIQC) was implemented prior to fMRI data pre-processing [55]. Scans were entered through a minimal preprocessing pipeline to obtain images and masks for Image Quality Metrics (IQMs): signal-to-noise ratio, framewise-displacement (FD), and segmented tissue summary statistics. The data from outliers on these metrics (>3 SD) were assessed visually. All scans were of high quality.

fMRI preprocessing was performed using fMRIPrep v. 20.2.1 [56]. The T1w image, which was preprocessed as described in the Supplementary Methods, was used for spatial normalization and alignment. For each BOLD-run per subject, the following preprocessing was performed. First, the initial first four volumes were non-steady state scans and were removed. Then, a reference volume and its skull-stripped version were generated and corrected for susceptibility distortions using fMRIPrep’s fieldmap-less approach. The BOLD reference was then co-registered to the T1w reference using bbregister (FreeSurfer v6.0.1) which implements boundary-based registration [57]. Co-registration of the BOLD reference to the T1 reference was configured with nine degrees of freedom to account for distortions remaining in the BOLD reference. Head-motion parameters with respect to the BOLD reference (transformation matrices and size rotation and translation parameters) for later denoising were estimated using MCFLIRT (FSL 5.0.9 [58]) before any spatiotemporal filtering. Head motion correction was performed in the original, native space. The BOLD time series were resampled onto the FreeSurfer surface: “fsaverage” and then into standard space (MNI152NLin2009cAsym). Grayordinates files containing 91k samples were generated.

Next, denoising of the data was performed in Matlab (R2019a) using custom scripts to regress out the following noise regressors: five aCompCor principal components from both the segmented WM and CSF [59], 24 motion parameters (3 rotation, 3 translation, their derivatives and quadratic terms [60]; cosine filters (128 s cut off)—low-frequency signal drift—regressors, and spike regressors for each frame that exceeded a threshold of 0.5 mm FD [61, 62].

For functional connectivity analyses, we used Glasser et. al.’s 2016 multimodal parcellation (360 cortical areas [63]), in addition to the 19 anatomically defined subcortical areas from the HCP [64]. We constructed functional connectivity matrices by extracting the mean BOLD time series for these 379 areas and subsequently computing the *z*-transformed Pearson correlation coefficients between the time-courses of all areas. The resulting matrices were binarized with a proportional threshold of 15%, which is the median of an ideal cost range (approx. 0.01–0.30). This provides for optimized sparsity in the thresholded graph [65] and improved stability of measures as compared to absolute thresholds [66].

We used a graph theory approach to calculate nodal global efficiency, local efficiency, betweenness centrality, and participation coefficient using the Brain Connectivity Toolbox [67]. We used three measures of functional integration and segregation—nodal global efficiency, local efficiency, and betweenness centrality. The efficiency metrics, which are known to be powerful and biologically plausible measures of brain network function, quantify the shortest path length between a select node and all other nodes in the brain (global) or neighboring nodes (local), whereas the betweenness measure quantifies the number of paths that pass through a given node and thus assesses that node’s criticality in the network [67, 68]. Additionally, to assess centrality, we used the graph theory measure participation coefficient, which quantifies whether a node is facilitating modular segregation [67, 69]. Module assignments for computing participation coefficient were chosen a priori based on the same multimodal parcellation as used to define areas, [63] assigning each of Glasser et al.’s 22 regions and the 19 anatomically defined subcortical areas as a module. See Supplementary Methods for more details.

For each graph theory metric, a generalized linear model (GLM) regression analysis was run and corrected for multiple comparisons using an FDR-threshold of *p* < 0.05. Separate analyses were performed to investigate the subtype-specific neurobiological correlates of current non-medicinal substance use (predictor: number of days in the past 6 months) and current binge-drinking (predictor: number of days in the past 6 months; men: 5 or more drinks per day, women: 4 or more drinks per day). Results were visualized using Connectome Workbench (version 1.5.0) (https://www.humanconnectome.org/software/connectome-workbench [70]). To aid the discussion and interpretation of results, the 360 cortical areas were additionally assigned to large-scale resting-state networks by first matching each area to its Brodmann Area and then determining the corresponding Intrinsic Connectivity Network based on Laird et al. (Supplementary Fig. 1) [71].

## Results

### Phenotypic space

The Bartlett’s test of Sphericity (_*X*_^*2*^ = 21299.81, *p* < 0.001) and Kaiser–Meyer–Olkin (KMO) test (KMO = 0.86) indicated appropriateness of the phenotypic data for EFA. Across all participants, we extracted 12 latent factors (*p* < 0.05), thus reducing the 74-variable phenotypic space into a 12-dimensional latent phenotypic space, within which we then performed the subsequent subtyping analysis. The 12 extracted latent factors collectively accounted for 44.8% of the common variance (see Supplementary Fig. 2 for the scree plot from the parallel analysis and Supplementary Table 4 for the EFA results). EFA model fit indices indicated good factor separation (*RMSEA* = *0.046, Tucker-Lewis Index* = *0.81*) and each factor had the minimum requirement of three salient loadings each [72, 73]. The 12 factors, their labels and factor loadings are displayed in Supplementary Table 5.

**Fig. 1:**
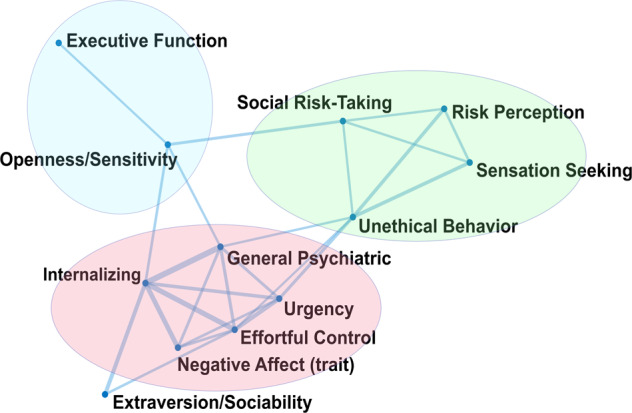
Spring-embedded network of 12 latent factors from EFA. In a spring-embedded network, nodes that are more strongly correlated are plotted closer together in two-dimensional space. Each node represents an EFA factor. Three functional domains emerged across all participants based on the underlying factor correlations: (1) an Approach-Related functional domain (green), (2) an Executive Function functional domain (blue), and (3) a Negative Emotionality functional domain (red).

From the 12 extracted latent factors, three factors saliently (>|0.30|) loaded on both trait and state variables (*internalizing, effortful control, extraversion/sociability*), one factor saliently loaded only on variables derived from performed tasks (*executive function*), one factor saliently loaded only on state variables (*general psychiatric symptoms*) and the remaining seven factors saliently loaded only on trait variables (Supplementary Table 5). The spring-embedded plot derived from the underlying factor correlations demonstrated that the twelve latent factors self-organized into three hypothesized phenotypic functional domains (Fig. 1, see Supplementary Table 6 for the factor correlations). These domains were: (1) an approach-related behavior domain that included *social risk-taking*, *unethical behavior, sensation seeking*, and *risk perception*; (2) an executive function domain that consisted of the factors *executive function* and *openness/sensitivity*; and (3) a negative emotionality domain that encompassed *internalizing*, *general psychiatric symptoms*, *urgency*, *negative affect (trait)*, and *(lack of) effortful control*, see Fig. 1.

### Recovered subtypes

**Fig. 2:**
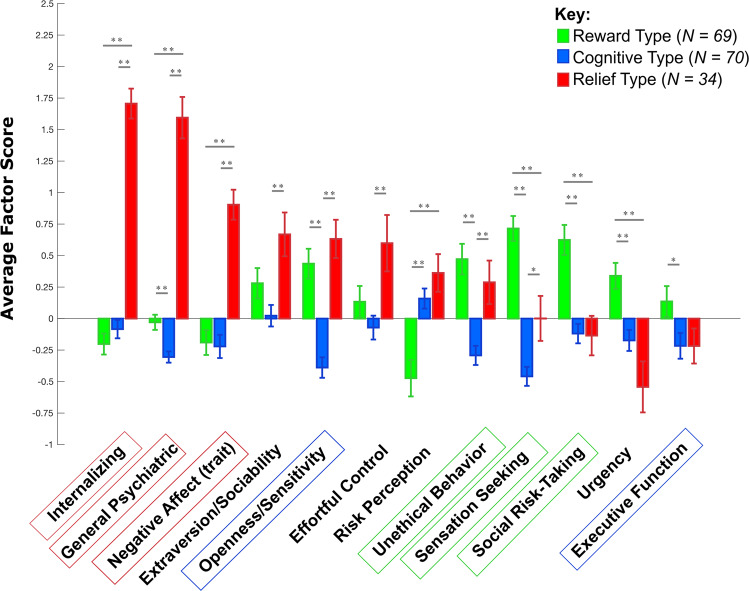
Phenotypic profiles of the 3 subtypes. Subtypes were determined using latent profile analysis on the EFA latent factors scores within individuals with past SUD diagnosis (*N* = 173). Three subtypes were recovered, which included a Reward Type with higher sensation seeking, social risk-taking, and unethical behavior (green); a Cognitive Type with lower openness/sensitivity and a lower executive function (blue), and a Relief Type with higher internalizing, general psychiatric symptoms, and negative affect (trait) (red). Independent samples t-test were done to compare the subtypes statistically (*p* < 0.05*, *p* < 0.01**). Error bars are standard error.

The LPA performed within this 12-dimensional phenotypic space and within participants with past SUD found that three subtypes existed among those with past SUD. BLRT was significant up to a 5-cluster solution (*p*_FDR_ < 0.05), however, the 3-cluster solution was chosen as the best model based on how the 3-cluster solution exceeded the second-best model of a 4-cluster solution (delta-BIC = 7.32). Demographic characteristics, clinical and substance use data for the recovered subtypes are described in Supplementary Tables 7a, b. The subtypes differed from each other in their phenotypic profiles (*p* < 0.05, Supplementary Table 8), with very large effect sizes for differences in the factors that best differentiated between subtypes (Cohen’s *D* = 1.7–2.8; Supplementary Table 9). We found (1) a “Reward type” with higher *sensation-seeking*, *social risk-taking*, and *unethical behavior*; (2) a “Cognitive type” with lower *openness/sensitivity* and lower performance on *executive function* tasks; (3) and a “Relief type” with high *internalizing* and *general psychiatric symptoms* and higher *negative affect (trait)* (see Fig. 2 for the phenotypic profiles of these subtypes). Additional results confirmed that we found the same subtype-specific profiles when comparing each subtype to control participants (see Supplementary Table 10). Importantly, while these results confirmed that impairments on all three hypothesized domains are linked to addiction, each discovered SUD subtype was characterized by impairments on only one of the three domains.

Demographic and clinical data converged with these results such that the Reward type had significantly higher non-medicinal substance use (past 6 months) and greater frequency of urine screens positive for tetrahydrocannabinol (THC) on MRI day, the Cognitive type had lower levels of education, and the Relief type had a significantly higher rate of diagnosed internalizing disorders (Supplementary Table 7a). While none of the correlations between EFA scores and current (modest) substance use were significant after Holm-Bonferroni correction, before correction the Reward type showed a correlation of current non-medicinal substance use with *unethical behavior* (*r* = *0*.*24, p* = *0.05*), and the Cognitive type with *effortful control* (*r* = *0.32, p* < *0.05*). Finally, there was no difference in the distribution of past primary SUD diagnosis (*equal distribution, χ2* = *4.71, p* = *0.32*) or gender distribution (*χ2* = *3.44 p* = *0.18*) between the three recovered subtypes.

### Resting-state functional connectivity per subtype

Each subtype demonstrated unique functional connectivity patterns that were linked to current return to substance use (see Table 2). For individuals in the Reward type, non-medicinal substance use was associated with functional connectivity in the Value/Reward (ACC/mPFC), Ventral Frontoparietal (inferior frontal), and the Salience (insula, frontal opercular, ACC/mPFC) networks. For individuals in the Cognitive type, non-medicinal substance use was linked to functional connectivity in the Auditory (auditory association cortex), Parietal Association (paracentral lobular/mid-cingulate), Frontoparietal (superior parietal), and Salience (ACC/mPFC) networks. For individuals in the Relief type, non-medicinal substance use was associated with functional connectivity with non-medicinal substance use in the Parietal Association (paracentral lobular/mid-cingulate), higher visual (motion complex, neighboring visual cortex) and Salience (paracentral lobular/mid-cingulate, insular, frontal opercular) networks. Detailed results including the specific implicated areas, regions, affected graph theory measures, directions of effects, and FDR-corrected *p* values for each significant parcel are summarized in Table 2. To visualize results, areas were projected onto template brains by subtype, collapsing across graph theory metrics in Supplementary Fig. 3. These results converged partially with the analysis on resting-state functional connectivity alterations related to alcohol binge drinking (see Supplementary Table 11 for detailed results). In the Relief type, three networks (Higher Visual, Salience, Auditory) were linked to both binge drinking and non-medicinal substance use. Both Relief and Reward type uniquely demonstrated an involvement of the Motor Planning networks linked to binge drinking, but not non-medicinal substance use. There were no significant results for the Cognitive type related to binge drinking.

**Table 2:** fMRI functional connectivity graph theory results by subtype.

Resting state network (Laird et al., 2011)	Region (Glasser et al., 2016)	Area (Glasser et al., 2016)	Graph theory measure	Greater or less than	*p*_FDR_
Reward type					
Value/Reward	19. ACC/mPFC	R 10v	Local efficiency	↓	0.0426
Value/Reward	19. ACC/mPFC	L 10v	Local efficiency	↓	0.0302
Value/Reward	19. ACC/mPFC	L 10r	Local efficiency	↓	1.36E−04
Value/Reward	19. ACC/mPFC	L 10r	Participation coeff.	↓	7.90E−04
Value/Reward	19. ACC/mPFC	L a10p	Betweennness	↑	0.019
Ventral-Frontoparietal	21. Inferior frontal	L IFSp	Nodal global efficiency	↓	0.0111
Ventral-Frontoparietal	21. Inferior frontal	L IFSp	Local efficiency	↓	8.77E−04
Ventral-Frontoparietal	21. Inferior frontal	L IFSp	Participation coeff.	↓	1.00E−06
Ventral-Frontoparietal	21. Inferior frontal	L 45	Participation coeff.	↓	1.30E−03
Ventral-Frontoparietal	17. Inferior parietal	R IP1	Betweenness	↑	0.0269
Salience	12. Insular/frontal opercular	R AVI	Local efficiency	↓	0.0327
Salience	12. Insular/frontal opercular	L FOP5	Nodal global efficiency	↓	0.0124
Salience	19. ACC/mPFC	L p32	Participation coeff.	↓	0.0281
Visual Primary	18. PCC	L ProS	Local efficiency	↓	0.0014
Visual Primary	18. PCC	L ProS	Participation coeff.	↓	8.57E−04
Default mode	18. PCC	L d23ab	Local efficiency	↓	0.012
Auditory	14. Lateral temporal	L TE1a	Local efficiency	↓	0.0346
Dorsal-Frontoparietal	22. Dorsolateral prefrontal	L SFL	Local efficiency	↓	0.0338
Midbrain	Subcortical	Brainstem	Participation coeff.	↓	0.0338
Cognitive type					
Auditory	11. Association auditory	L STGa	Betweenness	↑	0.006
Auditory	11. Association auditory	R STSva	Local efficiency	↓	0.0327
Auditory	11. Association auditory	L STSva	Participation coeff.	↓	0.0346
Parietal Association	7. Paracentral lobular/mid cingulate	L 5mv	Betweenness	↑	0.0342
Parietal Association	7. Paracentral lobular/mid cingulate	R 5L	Betweenness	↑	0.006
Frontoparietal	16. Superior parietal	R 7Am	Betweenness	↑	0.0291
Frontoparietal	16. Superior parietal	R 7Pm	Betweenness	↑	0.0199
Salience	19. ACC/mPFC	L p24pr	Betweenness	↑	0.0211
Salience	19. ACC/mPFC	L p24	Betweenness	↑	0.0199
Limbic/Memory	14. Lateral temporal	L TE1m	Betweenness	↑	0.0199
Higher visual	15. TPOJ	R TPOJ3	Participation coeff.	↓	0.0346
Relief type					
Parietal Association	7. Paracentral lobular/mid cingulate	L 5L	Nodal global efficiency	↓	8.00E−04
Parietal Association	7. Paracentral lobular/mid cingulate	R 5L	Nodal global efficiency	↓	6.18E−06
Parietal Association	7. Paracentral lobular/mid cingulate	R 5L	Local efficiency	↓	8.00E−04
Parietal Association	7. Paracentral lobular/mid cingulate	R 5L	Participation coeff.	↓	2.43E−05
Parietal Association	7. Paracentral lobular/mid cingulate	R 5mv	Participation coeff.	↓	0.0035
Higher Visual	5. Motion complex	L V4t	Betweenness	↑	3.96E−04
Higher Visual	5. Motion complex	L LO3	Betweenness	↑	2.12E−05
Higher Visual	5. Motion complex	L MT	Betweenness	↑	3.60E−05
Higher Visual	4. Ventral stream visual	L VVC	Betweenness	↑	0.0436
Salience	7. Paracentral lobular/mid cingulate	R 24dd	Betweenness	↑	3.59E−02
Salience	12. Insular/frontal opercular	L PoI1	Betweenness	↑	1.26E−02
Limbic/Memory	14. Lateral temporal	R TE2p	Betweenness	↑	1.49E−02
Auditory	11. Association auditory	R STGa	Betweenness	↑	1.49E−02

See Supplementary Fig. 3 for visualization of networks on template brain.

## Discussion

The goal of this study was to characterize individual heterogeneity across three functional domains in individuals with past SUDs. Our results revealed three distinct addiction subtypes, with each subtype demonstrating impairments in only one of the three relevant functional domains. For individuals in the Reward type, who showed higher approach-related behavior (e.g., high *sensation seeking* and the highest current non-medicinal substance use), substance use was related to functional connectivity in networks underlying value and reward representation. For individuals in the Cognitive type, who demonstrated challenges in executive function (e.g., lower *executive function* performance and lower levels of education), substance use was related to functional connectivity in networks underlying cognition, such as the Frontoparietal network. For individuals in the Relief type, who demonstrated higher negative emotionality (e.g., more *internalizing* and *psychiatric symptoms*, and more frequent diagnosis of internalizing disorders), substance use was related to functional connectivity in visual association networks. Overall, these results support the existence of substantial individual differences in the three mechanisms that have been linked to addiction. Importantly, while previous work had revealed abundant evidence on heterogeneity in approach/reward behavior, executive function and negative emotionality in addiction by investigating each functional domain separately [74–82], this is the first study to show that there are subgroups with differential functional profiles across these three domains.

The first step of our analysis provided empirical evidence for the existence of a general phenotypic space with three separable functional domains: 1) approach-related behavior, 2) executive function, and 3) negative emotionality. These results therefore support previous research proposing these as three independent (separable) behavioral domains [15, 16, 83], which ultimately led to the conceptualization of these as Research Domain Criteria (RDoC) domains [84, 85]. Importantly, our results further provide empirical evidence for the equal relevance of all three functional domains in addiction. This is important, since consensus discussions on the relevance of the RDoC domains for explaining addiction have mainly focused on constructs from the approach-related “positive valence” domain (e.g., “reward valuation,” “reward learning”, “expectancy”, “action selection”, and “habit”) [86]. Our results, however, support the equal importance of the RDoC cognitive and negative valence systems in SUDs, in accordance with a wealth of research demonstrating the importance of lower executive function and increased negative emotionality as addiction persistence mechanisms [7, 8, 87].

Our results further expand on previous work on functional domains in AUD in several ways. First, we provide additional empirical evidence for the relevance of three data-derived functional domains shown to be relevant in AUD by Kwako and colleagues: (1) incentive salience, (2) executive function, and (3) negative emotionality [10, 88]. Kwako and colleagues used a similar approach as ours for deriving the functional domains (EFA), in a data set that included 43.5% individuals with AUD. Overall, they found functional domains that were more narrowly defined than ours, largely due to differences in the included measures. Kwako and colleagues selected measures based on their theoretical relevance to addiction models (personal correspondence with Dr. Kwako), whereas we used an agnostic approach and included all available phenotypic data. Therefore, their “incentive salience” domain was indexed by the self-reported drive to consume substances, whereas our approach-/reward behavior domain was defined by the broader constructs of reward seeking (e.g., *sensation seeking* and *social risk-taking*). And while their executive function domain included measures of impulsivity, conscientiousness, and perseverance, ours was indexed by the broader measures of *openness/sensitivity* and *executive function*, which loaded on tasks that assessed cognitive flexibility/shifting, attention shifting, planning and working memory [89–91]. Both their and our negative emotionality domains were very similar and included measures of negative affect, internalizing psychiatric symptoms and aggression. Further, in our analysis “effortful control” (e.g., lack of conscientiousness, and perseverance) was highly correlated to measures of negative emotionality, but not to the other measures of executive function, a finding which we have previously reported in a completely independent data set [92]. Overall, while our phenotypic space described a broader phenotypic space, our results align with Kwako’s empirically derived results, and hence provide additional empirical evidence for common addiction models (e.g., [8]) that propose the relevance of these three domains in addiction. Importantly, however, our results provide additional novel empirical evidence on the relevance of this framework beyond AUD, by demonstrating the existence of the same subtypes, with impairments in these three functional domains, independent of the primary substance of choice.

We found individual differences for both trait and state factors in our analysis. Each subtype was characterized by factors that assessed general, temperament or personality characteristics. Individuals in the Relief and Cognitive types were additionally characterized by the *internalizing*, *general psychiatric* and *executive function* factors, which loaded on behavioral tasks and self-report of current psychiatric symptom. Previous research has repeatedly shown a convergence between state and trait characteristics [93, 94]. Trait-like stable characteristics such as for example “anxious temperament” are highly correlated with and predictive of changes in psychiatric symptoms (e.g., anxiety, depression) over time [93, 94]. Executive function is influenced by a highly heritable (99%) common factor [95]. Both previous research and our current findings therefore support our hypothesis that there are significant stable, “trait-like” individual differences in addiction that are linked to differences in state variables and would hence be relevant in different phases such as onset, persistence, and recovery from addiction. We propose that our “trait-like” subtypes have clinical relevance, because these trait-like individual differences interact with more dynamic processes, such as for example gradual effects caused by chronic drug-use. For example, an individual with trait-level challenges in executive function would be more vulnerable to the additional loss of cognitive control induced by chronic drug use over time than an individual who does not have such trait-level challenges.

Our findings on subtype-specific brain function during resting-state mirrored our behavioral results. We found that each subtype demonstrated unique patterns of large-scale resting-state network connectivity underlying current substance use. Neural correlates of substance use were identified in the Value/Reward, Ventral Frontoparietal, and Salience networks for those in the Reward type. These three networks represent incentive motivational value [96], are involved in approach behavior [97–99], and allocation of attentional control [100, 101], in line with the greater approach-behavior observed in this subtype. Aberrant engagement of these networks has also repeatedly been shown to be correlated with craving and substance use frequency in SUDs [102, 103]. These findings may also reflect current non-medicinal substance use and greater frequency of positive THC urine screen on MRI day. For those in the Cognitive type, higher non-medicinal substance use mapped on to connectivity in the Auditory, Parietal Association, Frontoparietal, and Salience networks. The auditory network has been reported to be associated with cognitive impairment [104]. Furthermore, the Parietal Association, Frontoparietal and Salience networks have shown connectivity responses related to executive control dysfunction in addiction [103, 105, 106], converging with the finding of challenges in executive function observed in this subtype. For those in the Relief type, non-medicinal substance use and binge drinking mapped on to connectivity in Parietal Association, higher visual and Salience networks. These three networks are implicated in effortful control [12, 103, 107–109], have been shown to be hyper-engaged in those with higher levels of increased anxiety [109], and have greater connectivity due to “hyperscanning” of the environment [110, 111]. In summary, our analysis of resting-state brain function correlated with current non-medicinal substance use recovered distinct neurobiological features that fit the phenotypic profiles of each subtype.

It has been suggested—and we agree—that a better understanding of the substantial neural and behavioral heterogeneity within SUDs by means of subtyping could spur the development of personalized treatments and preventions, improve treatment effectiveness, and inform resource allocation [112–116]. Our results underscore the importance of this effort, by providing empirical evidence for substantial individual differences in the neurobehavioral impairments on three functional domains linked to addiction that can be targeted separately in a Personalized Medicine approach. Initial evidence on the improved efficacy of subtype-specific treatments comes from a series of studies in individuals with AUD. These studies demonstrated that a pharmacological treatment that blocks the brain’s “pleasure response” was efficacious only in a subgroup of patients that indicated that their main drinking motive was pleasure seeking, but was less effective in reducing drinking in individuals who drank to cope with negative affect [36, 112, 117]. Other efficacious subtype-specific targeted treatments for the Relief Type may be therapies that target emotion regulation [e.g., pharmacotherapies used in anxiety/depression [118]; cognitive reappraisal [109]; specialized cognitive-behavioral therapy for SUDs with comorbid mental health disorders [119]. In contrast, the Cognitive Type may be best treated with cognitive trainings [e.g., working memory training [120, 121], pharmacological cognitive enhancers [120] or neuromodulation therapies targeted at enhancing cognitive control [122, 123]. Finally, the Reward type may have the best outcomes with motivational approaches that refocus their reward-seeking to non-substance related and non-immediate rewards [124, 125]. In summary, there is increasing evidence that targeted treatments for SUDs indeed outperform non-targeted treatment options [36, 117, 119].

The current study is only an initial step into the investigation of subtypes in addiction. Future work refining the proposed subtypes and developing clinical tools to “diagnose” them is needed. A limitation of the current study is that participants with currently diagnosed SUD(s) were not included. Another limitation is that the data set did not include detailed clinical data, or follow-up clinical data. It was therefore not possible to directly link the observed individual differences to clinical outcomes within this study, though there is an abundance of previous research in clinical samples that have demonstrated the importance of individual differences in approach-/reward related behavior, executive function, and negative emotionality for clinical outcomes [4, 126–129].

## Conclusion

As hypothesized, the current results provide empirical evidence for substantial individual differences in the mechanisms underlying addiction, and the existence of “trait-like” subtypes that each had impairments on only one of three identified addiction-relevant functional domains. These subtypes were found to be “addiction-general,” as they were independent of the different primary substances of choice. These results call for updated addiction models that factor in how these trait-like individual differences interact with the mechanisms driving the escalation and persistence of substance use. Finally, if further substantiated, these results could guide the development of individualized behavioral, pharmacological, and brain-focused preventative and treatment approaches that take these individual differences into account.

## Supplementary information

Supplemental Materials

## Data Availability

All neuroimaging data are available for download through Amazon Web Services or NITRC and a data usage agreement is not required. Phenotypic data are available for download through the Longitudinal Online Research and Imaging System (LORIS) database and Collaborative Informatics and Neuroimaging Suite (COINS) databases upon approval of a data usage agreement with NKI. Details are provided at http://fcon_1000.projects.nitrc.org/indi/enhanced/access.html.

## Footnotes

1 Abbreviations: AUDIT Alcohol Use Disorders Identification Test, ASI Addiction Severity Index, AUDADIS-IV Alcohol Use Disorder and Associated Disabilities Interview Schedule-IV, SSADDA Semi-Structured Assessment for Drug Dependence and Alcoholism, DSM-IV Diagnostic and Statistical Manual of Mental Disorders-IV, ICD-10 International Statistical Classification of Diseases and Related Health Problems (10th revision).
