# Supplementary material for: Subtypes in addiction and their neurobehavioral profiles across three functional domains
Source: Transl Psychiatry. 2023 Apr 18;13:127. doi: 10.1038/s41398-023-02426-1 (PMC10113211; doi:10.1038/s41398-023-02426-1)
Supplement: Supplementary file 1 — Supplemental Materials [file 41398_2023_2426_MOESM1_ESM.pdf]

Subtypes in Addiction and Their Neurobehavioral Profiles across Three Functional Domains

Gunner Drossel[1,2], Leyla R. Brucar, MSc[2], Eric Rawls, PhD[2], Timothy J. Hendrickson, MSc[3,4], Anna Zilverstand, PhD[2,5] *

1) Graduate Program in Neuroscience, University of Minnesota, Minneapolis, MN, USA
2) Department of Psychiatry & Behavioral Sciences, University of Minnesota, Minneapolis, MN, USA
3) University of Minnesota Informatics Institute, University of Minnesota, Minneapolis, MN, USA
4) Masonic Institute for the Developing Brain, University of Minnesota, Minneapolis, MN, USA
5) Medical Discovery Team on Addiction, University of Minnesota, Minneapolis, MN, USA

* Corresponding author

Supplementary Materials

Supplementary Methods

Factor Analysis of Phenotypic Data

Factors were extracted using a maximum likelihood as calculated by the expectation-maximization algorithm, and an oblimin rotation was used to allow for correlated factors. EFA was calculated in R using the "psych" package[1]. Bartlett's test of sphericity[2] was used to ensure the correlation matrix was not random and the KMO statistic[3] was required to be above a minimum of .50. MATLAB's 'graph' object was used to visualize the full phi correlation matrix from the factor analysis. EFA was chosen over other data reduction schemes, such as PCA, because EFA accounts for error due to unreliability in measurement[4] unlike PCA[5]. Also, maximum likelihood extraction is robust against violation of distributional assumptions[6,7], therefore providing a strong technique for reduction of questionnaire and other behavioral data. Weighted least squares and principal factors solution factoring extraction resulted in the same number of factors suggested. Factor analysis conducts an eigenvalue decomposition of the correlation matrix among items and does not assume that the items are measured on similar scales. The use of oblimin rotation allows for correlated factors which is critical to data reduction over a large phenotypic variable space due to how many factors are expected to be separable but closely related. Monte Carlo simulation was calculated using the "psych" package for R[1]. Other available methods for choosing the number of latent factors in a solution are not robust against violation of distributional assumptions, whereas permutation analysis ensures that this selection is not contingent on assumptions regarding underlying distributional qualities.

Anatomical fMRI Data Preprocessing*:*

For each subject's T1-weighted (T1w) image, anatomical preprocessing was performed. The T1w image was corrected for intensity non-uniformity (INU) using N4BiasFieldCorrection[8], distributed with Advanced Normalization Tools (ANTs) 2.3.3[9] and used as a T1w-reference throughout the remaining workflow. This T1w-reference was skull-stripped with a Nipype implementation[10] of the antsBrainExtraction.sh workflow (ANTs) using the OASIS30ANTs template. Brain tissue segmentation of CSF, WM, and GM was performed on the brain-extracted T1w image using FSL 5.0.9 (FSL, RRID:SCR_002823)[11]. Brain surfaces were reconstructed using recon-all (FreeSurfer v6.0.1, RRID:SCR_001847)[12], and the brain mask estimated previously was refined with a custom variation of the method to reconcile ANTs-derived and FreeSurfer-derived segmentations of the cortical gray-matter of Mindboggle (RRID:SCR_002438)[13]. Volume-based spatial normalization to the standard Montreal Neurological Institute (MNI) space as performed through nonlinear registration with antsRegistration (ANTs 2.3.3), using brain-extracted versions of both T1w reference and the T1w template. This template was used for spatial normalization: ICBM 152 Nonlinear Asymmetrical template version 2009c (TemplateFlow ID: MNI152NLin2009cAsym, RRID:SCR_008796)[14].

Graph Theory Measures

Graph theory efficiency metrics are more powerful and more biologically plausible for representing functional integration for small-world networks, such as brain networks, in comparison to other metrics from graph theory[15–17]. Nodal global efficiency, which is the average inverse shortest path length between a given node and all other nodes in the network, was used to quantify the importance of nodes for information transference[17]. Local efficiency measures information exchange with only the nearest neighbors of a node across a 'neighborhood', rather than all nodes in the brain[17]. Betweenness centrality is the fraction of all shortest paths in the network that pass through a given node, and it measures the extent to which a given brain region connects other regions in the network[17,18]. A node with high betweenness centrality would be denoted a 'bridging' node as it connects disparate parts of a network. Participation coefficient describes the centrality of a node by the ratio of its intramodular to intermodular connections and it thus quantifies whether a node is likely facilitating modular segregation[17]. Overall, these measures assess functional integration via nodal global and local efficiency and betweenness, and modular segregation via participation coefficient.

References:

1. Revelle, W. R. (2017). *psych: Procedures for Personality and Psychological Research*. https://www.scholars.northwestern.edu/en/publications/psych-procedures-for-personality-and-psychological-research

2. Bartlett, M. S. (1954). A Note on the Multiplying Factors for Various χ2 Approximations. *Journal of the Royal Statistical Society. Series B (Methodological)*, *16*(2), 296–298.

3. Kaiser, H. F. (1974). An index of factorial simplicity. *Psychometrika*, *39*(1), 31–36. https://doi.org/10.1007/BF02291575

4. Suhr, D. D. (2006). *Exploratory or confirmatory factor analysis?*

5. Bryant, F. B., & Yarnold, P. R. (1995). Comparing Five Alternative Factor-Models of the Student Jenkins Activity Survey: Separating the Wheat From the Chaff. *Journal of Personality Assessment*, *64*(1), 145–158. https://doi.org/10.1207/s15327752jpa6401_10

6. Fuller, E. L., & Hemmerle, W. J. (1966). Robustness of the maximum-likelihood estimation procedure in factor analysis. *Psychometrika*, *31*(2), 255–266. https://doi.org/10.1007/BF02289512

7. Muthen, B., & Kaplan, D. (1992). A comparison of some methodologies for the factor analysis of non-normal Likert variables: A note on the size of the model. *British Journal of Mathematical and Statistical Psychology*, *45*(1), 19–30. https://doi.org/10.1111/j.2044-8317.1992.tb00975.x

8. Tustison, N. J., Avants, B. B., Cook, P. A., Yuanjie, Z., Egan, A., Yushkevich, P. A., et al. (2010). N4ITK: Improved N3 Bias Correction. *IEEE Transactions on Medical Imaging*, *29*(6), 1310–1320. https://doi.org/10.1109/TMI.2010.2046908

9. Avants, B. B., Epstein, C. L., Grossman, M., & Gee, J. C. (2008). Symmetric diffeomorphic image registration with cross-correlation: Evaluating automated labeling of elderly and neurodegenerative brain. *Medical Image Analysis*, *12*(1), 26–41. https://doi.org/10.1016/j.media.2007.06.004

10. Gorgolewski, K., Burns, C. D., Madison, C., Clark, D., Halchenko, Y. O., Waskom, M. L., et al. (2011). Nipype: A Flexible, Lightweight and Extensible Neuroimaging Data Processing Framework in Python. *Frontiers in Neuroinformatics*, *5*. https://doi.org/10.3389/fninf.2011.00013

11. Zhang, Y., Brady, J. M., & Smith, S. M. (2011). *An HMRF-EM Algorithm for Partial Volume Segmentation of Brain MRI FMRIB Technical Report TR01YZ*.

12. Dale, A. M., Fischl, B., & Sereno, M. I. (1999). Cortical Surface-Based Analysis – I. Segmentation and Surface Reconstruction. *Neuroimage*, *9*, 179–194.

13. Klein, A., Ghosh, S. S., Bao, F. S., Giard, J., Häme, Y., Stavsky, E., et al. (2017). Mindboggling morphometry of human brains. *PLOS Computational Biology*, *13*(2), e1005350. https://doi.org/10.1371/journal.pcbi.1005350

14. Fonov, V., Evans, A., McKinstry, R., Almli, C., & Collins, D. (2009). Unbiased nonlinear average age-appropriate brain templates from birth to adulthood. *NeuroImage*, *47*, S102. https://doi.org/10.1016/S1053-8119(09)70884-5

15. Achard, S. (2006). A Resilient, Low-Frequency, Small-World Human Brain Functional Network with Highly Connected Association Cortical Hubs. *Journal of Neuroscience*, *26*(1), 63–72. https://doi.org/10.1523/JNEUROSCI.3874-05.2006

16. Latora, V., & Marchiori, M. (2003). Economic small-world behavior in weighted networks. *The European Physical Journal B - Condensed Matter and Complex Systems*, *32*(2), 249–263. https://doi.org/10.1140/epjb/e2003-00095-5

17. Rubinov, M., & Sporns, O. (2010). Complex network measures of brain connectivity: Uses and interpretations. *NeuroImage*, *52*(3), 1059–1069. https://doi.org/10.1016/j.neuroimage.2009.10.003

18. Freeman, L. C. (1978). Centrality in social networks conceptual clarification. *Social Networks*, *1*(3), 215–239. https://doi.org/10.1016/0378-8733(78)90021-7

Supplementary Figures

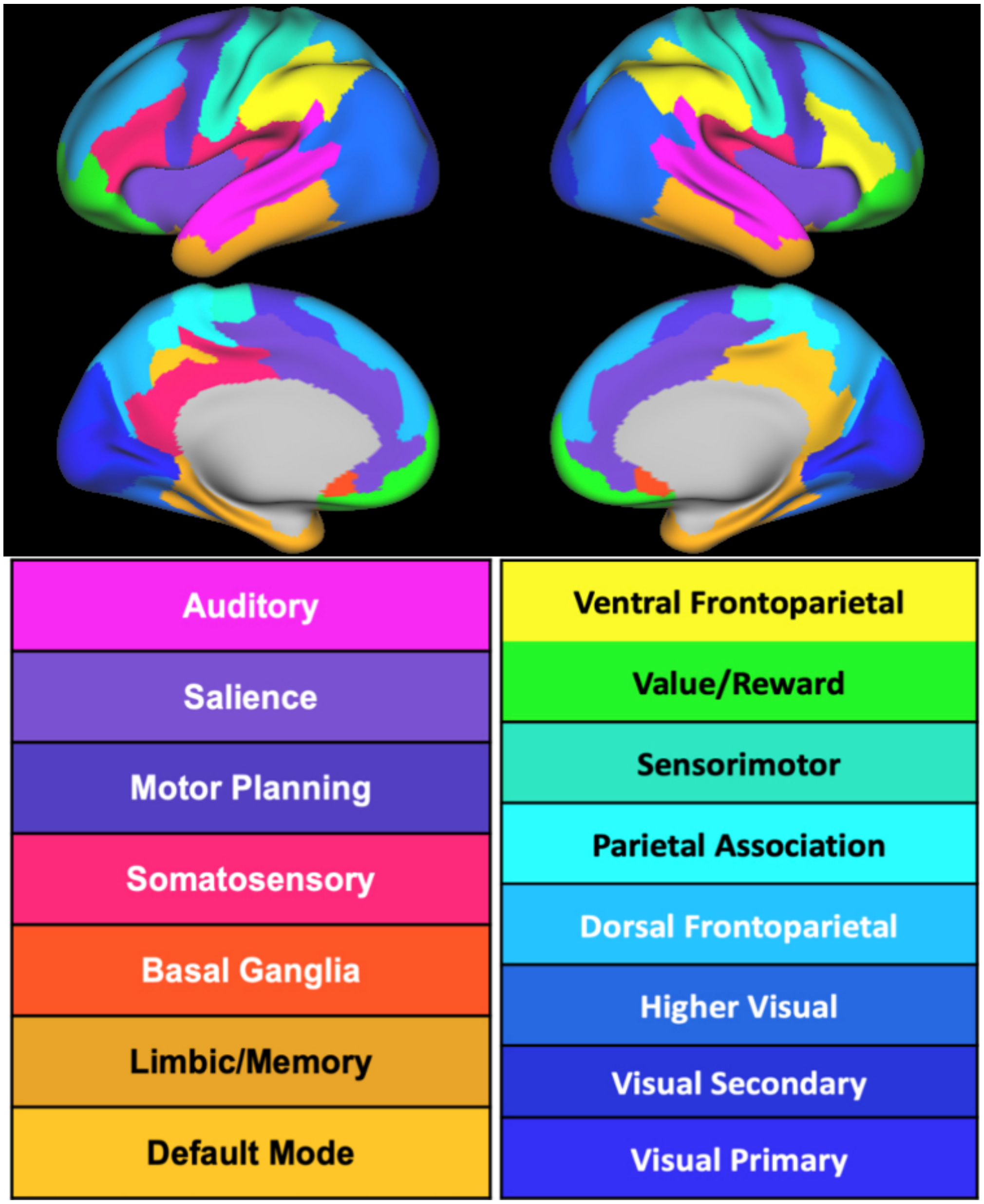

Supplementary Figure 1: Large-scale resting-state networks separately colored. To aid the discussion and interpretation of results, each of the 360 cortical areas from the Glasser et al., 2016 template were assigned into one of fifteen Intrinsic Connectivity Networks described by Laird et al., 2011 by matching each region to its Brodmann Area.

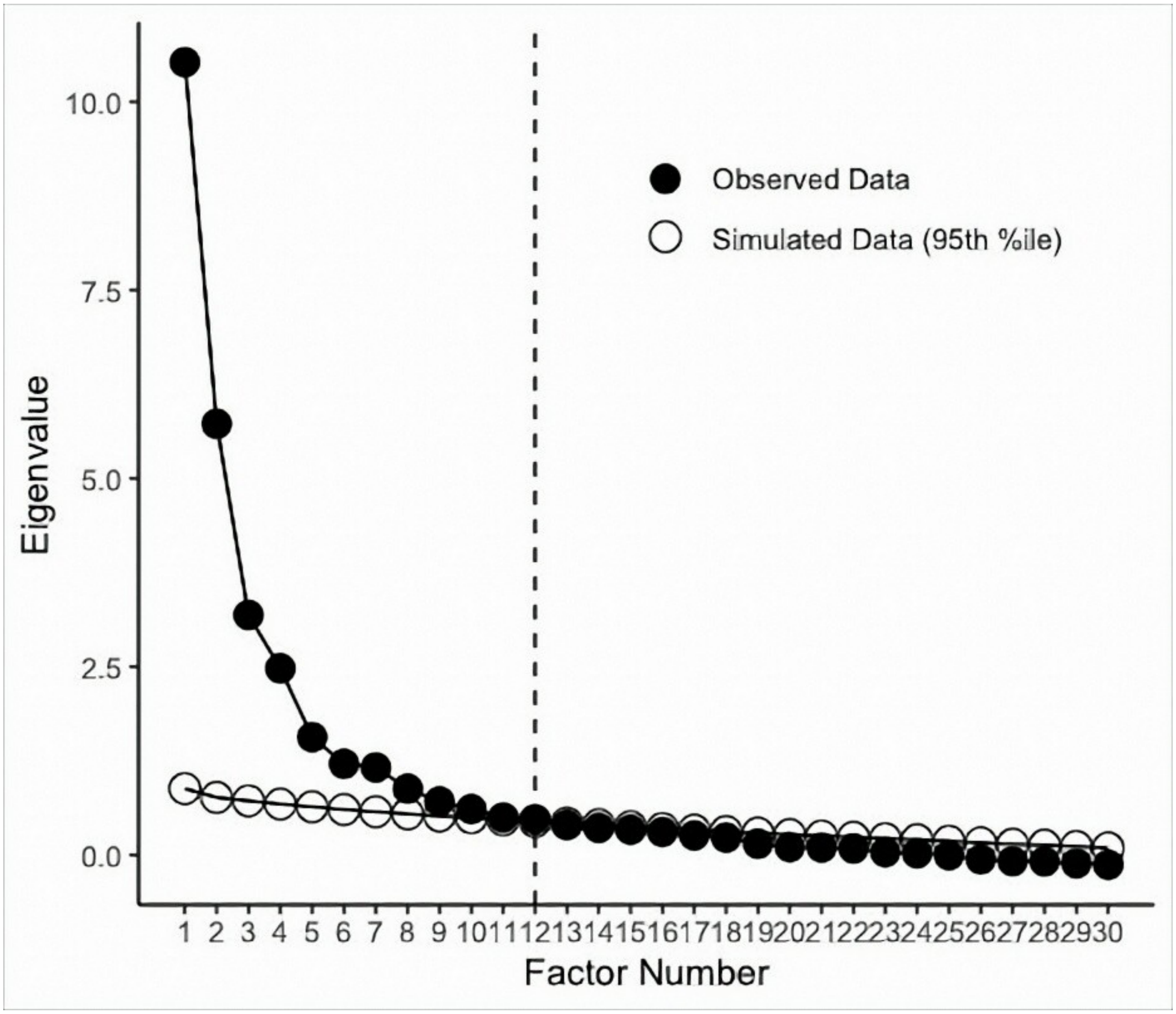

Supplementary Figure 2: Scree plot for visualization and interpretation of the results from the Monte Carlo simulation (parallel analysis) with eigenvalues (a metric of variance explained) for each factor extractable from the analysis. We retained the number of factors with observed eigenvalues that are larger than those extracted from corresponding factors based on simulated data. Here, it is 12 latent factors.

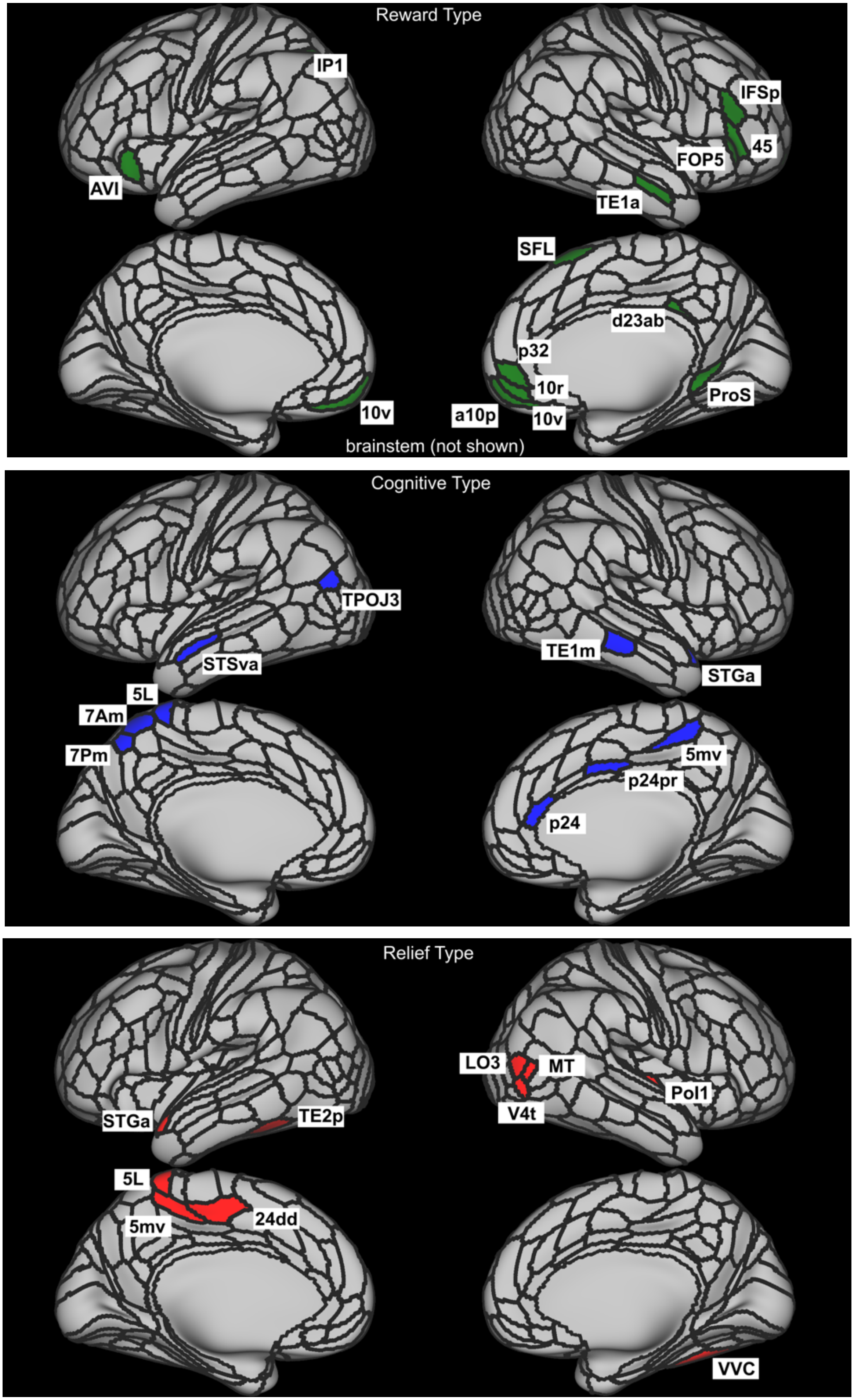

Reward Type
IP1
AVI
IFSp
45
FOP5
TE1a
SFL
d23ab
p32
10r
10v
a10p
10v
ProS
brainstem (not shown)
Cognitive Type
TPOJ3
STSva
TE1m
STGa
5L
7Am
7Pm
5mv
p24pr
p24
Relief Type
STGa
TE2p
LO3
MT
PoI1
V4t
5L
5mv
24dd
VVC

Supplementary Figure 3: Graph theory results per subtypes were projected onto a template brain. The area borders denote the 360 cortical areas from Glasser et al., 2016. Colored areas correspond to areas showing significant alterations linked to current non-medicinal substance use on any of the investigated graph theory measures (see Table 2, areas are labelled based on Glasser et al., 2016). *Top (Green): Reward type; Middle (Blue): Cognitive Type; Bottom (Red): Relief Type*. ***Abbreviations****: AVI: Anterior Ventral Insular Area, FOP5: Area Frontal Opercular 5, IFSp: Inferior Frontal Sulcus posterior, IP1: Area IntraParietal 1, LO3: Area Lateral Occipital 3, MT: Middle Temporal Area, SFL: Superior Frontal Language Area, TE1a: Area temporal 1 anterior, TE1m: Area temporal 1 medial, TE2p: Area temporal 2 posterior, TPOJ3: Area TemporoParietoOccipital Junction 3, VVC: Ventral Visual Complex. a = anterior, d = dorsal, p = posterior, v = ventral pr = prime. Numbers correspond to Brodmann areas.*

Supplementary Tables

Supplementary Table 1: Diagnoses for Past Multiple Use Disorder Group	
**Past Substance Use Disorder Combinations**	**N (individuals)**
Alcohol, Cannabis	29
Alcohol, Cannabis, Cocaine	13
Alcohol, Cocaine	7
Alcohol, Cannabis, Hallucinogen	3
Alcohol, Cannabis, Cocaine, Amphetamine	2
Cocaine	2
Alcohol, Amphetamine, Hallucinogen	1
Alcohol, Cannabis, Amphetamine	1
Alcohol, Hallucinogen	1
Alcohol, Opioid	1
Alcohol, Opioid, Cocaine, Amphetamine	1
Alcohol, Polysubstance	1
Amphetamine	1
Cannabis, Cocaine	1
Cannabis, Cocaine, Opioid, Sedative/Hypnotic	1
Cannabis, Inhalant	1
Cannabis, Phencyclidine, Cocaine	1
Cannabis, Sedative/Hypnotic	1

Supplementary Table 2: Items Included in the Exploratory Factor Analysis	
**Variable**	**Item Description**
ant_01	Attention Network Test: Alert Effect (task)
ant_02	Attention Network Test: Orienting Effect (task)
ant_03	Attention Network Test: Conflict Effect (task)
asrm_148	ASR Anxious/Depressed (past 6 months)
asrm_149	ASR Withdrawn (past 6 months)
asrm_150	ASR Somatic Complaints (past 6 months)
asrm_151	ASR Thought Problems (past 6 months)
asrm_152	ASR Attention Problems (past 6 months)
asrm_153	ASR Aggressive Behavior (past 6 months)
asrm_154	ASR Rule-breaking (past 6 months)
asrm_155	ASR Intrusive (past 6 months)
atq_78	ATQ Fear
atq_79	ATQ Frustration
atq_80	ATQ Sadness
atq_81	ATQ Discomfort

atq_83	ATQ Activation Control
atq_84	ATQ Attentional Control
atq_85	ATQ Inhibitory Control
atq_87	ATQ Sociability
atq_88	ATQ High Intensity Pleasure
atq_89	ATQ Positive Affect
atq_91	ATQ Neutral Perceptual Sensitivity
atq_92	ATQ Affective Perceptual Sensitivity
atq_93	ATQ Associative Sensitivity
dbdi_22	Becks Depression Inventory Total (past 2 weeks)
cogfq_26	Cognitive Failures Questionnaire Total Score (past 6 months)
dkefscwi_22	DKEFS Color Word Interference: Inhibition vs. Combined Naming + Reading Scaled (task)
df_19	DKEFS Design Fluency: Combined Filled + Empty Dots: Composite (task)
df_21	DKEFS Design Fluency: Switching vs. Combined Filled Contrast (task)
tow_47	DKEFS Tower: Total Achievement Scaled (task)
tow_49	DKEFS Tower: Total Rule Violation Scaled (task)
dkefstmt_24	DKEFS Trail Making: Combined Number + Letter Sequencing Composite (task)
dkefstmt_32	DKEFS Trail Making: Switching vs. Combined Number + Letter Sequencing Scaled (task)
vf_37	DKEFS Verbal Fluency: Letter Fluency vs. Category Fluency Scaled (task)
vf_39	DKEFS Verbal Fluency: Category Switching vs. Category Fluency Scaled (task)
dosp_091	DOSPERT Ethical Scale (Risk Taking)
dosp_092	DOSPERT Financial Scale (Risk Taking)
dosp_093	DOSPERT Health/Safety Scale (Risk Taking)
dosp_094	DOSPERT Recreational Scale (Risk Taking)
dosp_095	DOSPERT Social Scale (Risk Taking)
dosp_097	DOSPERT Ethical Scale (Risk Perception)
dosp_098	DOSPERT Financial Scale (Risk Perception)
dosp_099	DOSPERT Health/Safety Scale (Risk Perception)
dosp_100	DOSPERT Recreational Scale (Risk Perception)
dosp_101	DOSPERT Social Scale (Risk Perception)
dosp_103	DOSPERT Ethical Scale (Expected Benefits)
dosp_104	DOSPERT Financial Scale (Expected Benefits)
dosp_105	DOSPERT Health/Safety Scale (Expected Benefits)
dosp_106	DOSPERT Recreational Scale (Expected Benefits)
dosp_107	DOSPERT Social Scale (Expected Benefits)
neoffi_68	NEO Neuroticism
neoffi_69	NEO Extraversion
neoffi_70	NEO Openness

neoffi_72	NEO Agreeableness
neoffi_73	NEO Conscientiousness
penncnp_0067	Penn CNP Conditional Exclusion Test (Efficiency) (task)
penncnp_0108	Penn CNP Penn Word Memory Test (Total Correct Responses) (task)
penncnp_0136	Penn CNP Penn Emotion Recognition Test: Correct Anger Identifications (task)
penncnp_0137	Penn CNP Penn Emotion Recognition Test: Correct Fear Identifications (task)
penncnp_0138	Penn CNP Penn Emotion Recognition Test: Correct Happy Identifications (task)
penncnp_0140	Penn CNP Penn Emotion Recognition Test: Correct Sad Identifications (task)
penncnp_0210	Penn CNP Face Memory Task (Total Correct Responses) (task)
penncnp_0238	Penn CNP Penn Short Visual Object Learning Test (Efficiency) (task)
dseq_47	STAI State Anxiety ("present feelings")
dseq_48	STAI Trait Anxiety ("how you generally feel")
tsc40_47	Trauma Symptom Checklist (past 2 months)
upps_60	UPPS-P Impulsive Behavior Scale: Negative Urgency
upps_61	UPPS-P Impulsive Behavior Scale: Lack of Premeditation
upps_62	UPPS-P Impulsive Behavior Scale: Lack of Perseverance
upps_63	UPPS-P Impulsive Behavior Scale: Sensation Seeking
upps_64	UPPS-P Impulsive Behavior Scale: Positive Urgency
int_12	WASI Verbal Comprehension Index Composite (task)
int_13	WASI Perceptual Reasoning Index Composite (task)
wiat_08	WIAT Composite Standard (task)

Abbreviations:
ASR: Adult Self Report; ATQ: Adult Temperament Questionnaire; DKEFS: Delis-Kaplan Executive Functioning System; DOSPERT: Domain Specific Risk Taking Scale; NEO: Neo Five Factor Inventory; Penn CNP: Penn Computerized Neurocognitive Battery; STAI: State Trait Anxiety Inventory; UPPS-P: UPPS-P Impulsive Behavior Scale, WASI: Wechsler Abbreviated Scale Intelligence, WIAT: Wechsler Individual Achievement Test

Supplementary Table 3: Results urine screen before MRI						
	**Reward Type**	**Controls matched to Reward Type**	**Cognitive Type**	**Controls matched to Cognitive Type**	**Relief Type**	**Controls matched to Relief Type**
	N=37<br>(N=2 missing data)	N=35<br>(N=4 missing data)	N=38<br>(N=7 missing data)	N=43<br>(N=2 missing data)	N=16<br>(N=4 missing data)	N=19<br>(N=1 missing data)
Benzodiazepines	0 (0.0%)	**1 (2.9%)**	**2 (5.3%)**	0 (0.0%)	**1 (6.3%)**	0 (0.0%)
Tetrahydrocannabinol (THC)	**11 (29.7%)**	**2 (5.7%)**	**4 (10.5%)**	**3 (7.0%)**	**1 (6.3%)**	**2 (10.5%)**
Phencyclidine	0 (0.0%)	0 (0.0%)	**1 (2.6%)**	0 (0.0%)	0 (0.0%)	0 (0.0%)
Amphetamine	0 (0.0%)	0 (0.0%)	0 (0.0%)	**1 (2.3%)**	0 (0.0%)	0 (0.0%)
Barbiturates	0 (0.0%)	0 (0.0%)	0 (0.0%)	0 (0.0%)	0 (0.0%)	**1 (5.3%)**
Cocaine	0 (0.0%)	0 (0.0%)	**1 (2.6%)**	0 (0.0%)	**1 (6.3%)**	0 (0.0%)
Methadone	0 (0.0%)	0 (0.0%)	0 (0.0%)	0 (0.0%)	0 (0.0%)	0 (0.0%)
Opiates	0 (0.0%)	0 (0.0%)	**1 (2.6%)**	0 (0.0%)	0 (0.0%)	0 (0.0%)
Methampetamine	0 (0.0%)	0 (0.0%)	0 (0.0%)	0 (0.0%)	0 (0.0%)	0 (0.0%)
Oxycodone	0 (0.0%)	0 (0.0%)	**1 (2.6%)**	0 (0.0%)	0 (0.0%)	0 (0.0%)
Ecstasy	0 (0.0%)	0 (0.0%)	0 (0.0%)	0 (0.0%)	0 (0.0%)	0 (0.0%)

Supplementary Table 4: Percent Variance, Cumulative Variance, and Eigenvalues per Factor

	Internalizing	General Psychiatric	Effortful Control	Executive Function	Sensation Seeking	Unethical Behavior	Urgency	Openness/ Sensitivity	Extraversion/ Sociability	Risk Perception	Negative Affect (Trait)	Social Risk-Taking
SS Loadings (eigenvalues)	4.15	3.62	3.49	3.28	2.99	2.82	2.49	2.27	2.14	2.10	2.05	1.73
Proportion Variance	0.06	0.05	0.05	0.04	0.04	0.04	0.03	0.03	0.03	0.03	0.03	0.02
Cumulative Variance	0.06	0.10	0.15	0.20	0.24	0.28	0.31	0.34	0.37	0.40	0.42	0.45
Proportion Explained	0.13	0.11	0.11	0.10	0.09	0.09	0.08	0.07	0.06	0.06	0.06	0.05
Cumulative Proportion	0.13	0.23	0.34	0.44	0.53	0.61	0.69	0.76	0.82	0.89	0.95	1.00

Root Mean Squared Error of Approximation (RMSEA)	0.046
90% Confidence Intervals	0.044 and 0.048
Tucker-Lewis Index (TLI)	0.814
Bayesian Information Criteria (BIC)	-7752.52
Fit based upon off diagonal values	0.97
Common variance explained	0.448

Supplementary Table 5: Loading Values (pattern matrix) for 12 Factors from EFA												
	Internalizing	General Psychiatric	Effortful Control	Executive Function	Sensation Seeking	Unethical Behavior	Urgency	Openness/ Sensitivity	Extraversion/ Sociability	Risk Perception	Negative Affect (Trait)	Social Risk-Taking
STAI Trait Anxiety ("how you generally feel")	**0.85**	0.00	0.11	0.00	0.03	0.02	0.06	0.02	-0.09	0.01	-0.01	0.01
STAI State Anxiety (“present feelings”)	**0.80**	-0.02	0.03	-0.03	-0.02	0.00	0.11	-0.01	0.00	0.01	-0.02	-0.01
Becks Depression Inventory Total (past 2 weeks)	**0.57**	0.28	-0.01	-0.08	0.00	0.06	-0.05	0.01	-0.03	0.06	0.16	0.01
NEO Neuroticism	**0.52**	0.01	0.15	-0.04	0.04	0.00	0.21	0.07	-0.15	-0.06	0.13	-0.07
ASR Anxious/Depressed (past 6 months)	**0.51**	**0.51**	0.03	-0.01	0.01	0.04	-0.06	0.09	-0.08	0.02	-0.01	-0.09
Trauma Symptom Checklist (past 2 months)	**0.49**	0.20	-0.02	0.03	-0.03	0.08	0.08	0.13	-0.06	0.11	0.20	0.06
ATQ Sadness	**0.41**	-0.02	-0.04	0.11	-0.09	-0.09	0.05	0.26	0.06	0.01	0.28	-0.04
ASR Thought Problems (past 6 months)	-0.06	**0.68**	0.00	-0.02	0.08	-0.03	0.10	0.18	-0.09	-0.03	0.06	-0.01
ASR Aggressive Behavior (past 6 months)	0.20	**0.67**	-0.06	0.05	-0.01	-0.04	0.12	-0.11	0.07	-0.06	0.08	0.03
ASR Withdrawn (past 6 months)	0.10	**0.61**	0.02	-0.05	0.05	0.09	-0.07	0.01	**-0.41**	0.05	-0.03	0.02
ASR Rule-breaking (past 6 months)	0.06	**0.54**	0.18	-0.02	-0.05	0.10	0.21	0.03	0.09	0.06	-0.13	0.13
ASR Intrusive (past 6 months)	-0.08	**0.54**	0.07	0.16	-0.05	0.04	0.21	-0.02	**0.32**	0.00	-0.02	0.03
ASR Somatic Complaints (past 6 months)	0.12	**0.53**	0.03	-0.03	-0.06	-0.05	-0.05	0.10	-0.01	-0.05	0.20	0.00
ASR Attention Problems (past 6 months)	0.11	**0.53**	**0.52**	0.00	0.00	-0.01	-0.09	0.05	0.03	0.05	0.05	-0.01
NEO Conscientiousness	-0.02	0.00	**-0.83**	-0.02	-0.01	0.04	-0.06	0.03	0.09	0.01	0.04	-0.07
UPPS-P Impulsive Behavior: Lack of Perseverance	0.08	-0.02	**0.82**	-0.05	-0.04	0.05	0.05	0.02	-0.02	-0.04	-0.07	-0.06
ATQ Activation Control	0.03	-0.02	**-0.72**	-0.02	-0.02	-0.10	0.09	0.03	0.03	0.00	-0.19	0.01
ATQ Attentional Control	-0.04	-0.02	**-0.46**	-0.07	-0.06	-0.01	-0.09	-0.07	0.01	-0.03	**-0.40**	0.10
UPPS-P Impulsive Behavior: Lack of Premeditation	-0.01	-0.11	**0.41**	0.12	0.00	-0.05	**0.35**	-0.09	0.14	-0.10	-0.20	0.05
Cognitive Failures Questionnaire Total Score (past 6 months)	0.19	0.03	**0.37**	0.03	0.03	-0.04	0.12	0.05	0.02	0.04	0.28	0.19
DKEFS Verbal Fluency: Letter Fluency vs. Category Fluency Scaled (task)	-0.03	0.01	0.13	-0.02	-0.04	-0.03	-0.08	-0.03	-0.02	0.02	0.06	0.10

WASI Perceptual Reasoning Index Composite (task)	-0.06	0.04	-0.02	**0.72**	0.13	0.03	-0.01	-0.02	-0.05	0.03	0.02	0.03
WASI Verbal Comprehension Index Composite (task)	0.01	-0.01	0.05	**0.58**	-0.01	0.06	-0.06	0.21	-0.02	-0.11	-0.09	0.06
WIAT Composite Standard (task)	0.17	0.02	-0.01	**0.52**	-0.07	0.03	-0.08	-0.02	0.08	-0.02	0.02	0.00
DKEFS Design Fluency: Combined Filled + Empty Dots: Composite (task)	0.00	0.08	-0.06	**0.46**	0.04	0.03	0.03	-0.02	0.15	-0.08	0.04	-0.02
Penn CNP Penn Word Memory Test (Total Correct Responses) (task)	-0.01	-0.08	0.06	**0.44**	-0.03	-0.05	-0.03	0.09	-0.04	0.04	-0.03	-0.03
DKEFS Tower: Total Achievement Scaled (task)	-0.07	0.04	-0.04	**0.43**	0.04	0.09	0.01	-0.11	-0.06	0.03	0.09	0.10
DKEFS Trail Making: Combined Number + Letter Sequencing Composite (task)	0.01	0.00	-0.09	**0.42**	0.00	-0.03	-0.04	-0.09	0.17	-0.08	0.13	0.02
Penn CNP Penn Short Visual Object Learning Test (Efficiency) (task)	-0.10	-0.05	0.07	**0.39**	0.06	0.14	-0.06	0.09	-0.04	0.02	0.02	0.01
Penn CNP Face Memory Task (Total Correct Responses) (task)	0.04	-0.09	0.06	**0.38**	-0.08	-0.06	-0.04	0.13	0.03	-0.06	0.01	0.07
Penn CNP Conditional Exclusion Test (Efficiency) (task)	-0.14	0.02	0.05	**0.38**	0.14	0.03	0.03	-0.05	-0.12	0.09	0.07	0.05
Attention Network Test: Conflict Effect (task)	0.10	0.02	-0.05	**-0.38**	-0.10	-0.09	-0.02	0.03	0.15	-0.07	-0.03	-0.01
DKEFS Tower: Total Rule Violation Scaled (task)	-0.03	0.00	-0.02	**0.34**	0.00	0.07	-0.04	-0.05	-0.08	0.07	-0.02	0.19
Penn CNP Penn Emotion Recognition Test: Correct Fear Identifications (task)	-0.17	0.03	0.06	0.18	0.04	-0.02	0.02	0.17	-0.09	-0.01	0.02	-0.04
Attention Network Test: Alert Effect (task)	-0.05	-0.03	0.03	-0.16	0.04	-0.07	0.03	0.02	-0.02	-0.04	0.02	0.03
DKEFS Trail Making: Switching vs. Combined Number + Letter Sequencing Scaled (task)	-0.01	0.06	0.00	0.15	-0.09	0.03	0.09	0.04	-0.13	0.11	-0.08	0.03
Penn CNP Penn Emotion Recognition Test: Correct Sad Identifications (task)	-0.10	-0.01	0.05	0.12	0.03	-0.09	-0.08	0.06	-0.07	0.01	0.08	0.03
DOSPERT Recreational Scale (Risk Taking)	0.04	-0.02	0.03	0.04	**0.94**	-0.01	-0.04	-0.06	0.03	0.00	0.02	0.07

UPPS-P Impulsive Behavior Scale: Sensation Seeking	-0.09	0.05	-0.09	-0.04	**0.75**	0.10	0.14	0.07	0.08	0.04	-0.03	0.07
DOSPERT Recreational Scale (Expected Benefits)	0.06	-0.01	0.02	0.15	**0.65**	0.08	-0.01	0.18	-0.01	-0.09	-0.07	-0.03
ATQ High Intensity Pleasure	-0.04	0.11	0.06	-0.10	**0.30**	0.13	0.14	0.16	0.16	-0.01	-0.22	-0.02
DOSPERT Ethical Scale (Expected Benefits)	0.06	-0.05	-0.02	0.14	0.03	**0.80**	-0.02	0.02	-0.03	0.00	-0.02	-0.06
DOSPERT Ethical Scale (Risk Taking)	0.07	-0.01	0.08	-0.09	0.00	**0.66**	0.06	-0.11	0.09	-0.03	0.03	0.13
DOSPERT Health/Safety Scale (Expected Benefits)	-0.16	0.07	0.05	0.02	0.06	**0.58**	0.14	0.10	-0.06	-0.07	-0.07	-0.05
DOSPERT Financial Scale (Expected Benefits)	0.09	-0.12	0.01	-0.03	0.07	**0.44**	-0.05	0.06	0.09	0.02	0.05	0.15
DOSPERT Health/Safety Scale (Risk Taking)	-0.01	0.02	0.05	-0.13	0.23	**0.41**	0.18	-0.04	-0.01	-0.10	0.00	0.23
Penn CNP Penn Emotion Recognition Test: Correct Happy Identifications (task)	0.07	-0.05	0.06	0.06	0.07	-0.19	-0.08	-0.01	0.02	0.00	-0.02	0.07
Attention Network Test: Orienting Effect (task)	0.05	-0.02	-0.01	-0.04	0.05	-0.07	0.00	0.06	0.00	0.01	-0.04	0.06
UPPS-P Impulsive Behavior: Negative Urgency	0.20	0.03	0.05	0.02	-0.03	0.01	**0.76**	0.05	0.01	0.01	0.10	0.02
UPPS-P Impulsive Behavior: Positive Urgency	0.03	0.07	0.04	-0.14	0.11	0.08	**0.69**	0.05	-0.04	0.03	-0.04	-0.07
NEO Agreeableness	0.01	-0.06	0.10	0.02	-0.01	-0.13	**-0.39**	0.22	0.15	0.14	-0.12	-0.20
DKEFS Verbal Fluency: Category Switching vs. Category Fluency Scaled (task)	0.07	-0.01	0.01	-0.09	-0.07	-0.04	-0.10	-0.08	0.01	-0.02	0.04	0.04
ATQ Affective Perceptual Sensitivity	0.05	-0.02	-0.05	0.04	-0.07	-0.04	0.04	**0.65**	0.04	0.01	0.10	0.02
NEO Openness	0.01	0.03	0.01	0.06	0.10	0.02	-0.01	**0.62**	0.06	-0.04	-0.11	0.18
ATQ Associative Sensitivity	-0.02	0.09	0.08	-0.01	0.12	0.08	0.12	**0.50**	-0.02	0.04	0.16	0.16
ATQ Neutral Perceptual Sensitivity	0.04	0.08	-0.18	-0.06	-0.05	-0.05	-0.06	**0.45**	0.12	-0.03	0.03	0.05
DKEFS Design Fluency: Switching vs. Combined Filled Contrast (task)	-0.02	0.00	-0.02	0.00	0.03	0.00	-0.06	-0.12	-0.10	0.08	0.08	0.08
NEO Extraversion	-0.14	0.04	-0.13	-0.07	0.13	0.04	-0.05	0.04	**0.77**	0.03	0.02	-0.04
ATQ Sociability	0.04	-0.04	0.02	0.02	-0.01	-0.02	0.01	0.04	**0.73**	0.04	-0.06	0.05
ATQ Positive Affect	**-0.31**	-0.08	-0.02	0.00	0.00	-0.06	-0.01	0.20	**0.31**	0.03	0.00	0.09
Penn CNP Penn Emotion Recognition Test: Correct Anger Identifications (task)	-0.08	0.04	0.03	0.12	0.07	-0.05	0.02	0.12	-0.15	-0.03	0.04	-0.04

DOSPERT Health/Safety Scale (Risk Perception)	0.09	0.01	-0.01	-0.04	-0.01	-0.25	-0.13	0.01	0.11	**0.63**	0.00	0.00
DOSPERT Recreational Scale (Risk Perception)	-0.04	0.03	-0.04	-0.07	**-0.50**	0.09	0.00	-0.01	0.04	**0.62**	0.03	0.13
DOSPERT Financial Scale (Risk Perception)	0.00	-0.04	-0.06	0.17	-0.02	-0.05	0.10	-0.04	-0.04	**0.56**	-0.02	-0.14
DOSPERT Social Scale (Risk Perception)	0.07	0.01	0.03	-0.05	0.17	0.12	0.03	-0.02	0.03	**0.54**	0.07	**-0.38**
DOSPERT Ethical Scale (Risk Perception)	0.00	-0.06	-0.02	-0.14	0.10	**-0.46**	0.06	0.05	-0.01	**0.53**	-0.08	-0.02
ATQ Discomfort	0.07	0.01	-0.01	0.08	-0.05	-0.07	0.03	0.13	-0.17	-0.02	**0.54**	0.11
ATQ Fear	0.18	-0.04	0.15	0.04	-0.21	0.02	0.09	0.19	-0.05	0.07	**0.42**	-0.09
ATQ Frustration	0.10	0.11	0.01	0.09	-0.07	0.09	0.23	-0.13	0.06	-0.09	**0.40**	0.01
ATQ Inhibitory Control	0.13	-0.07	-0.14	0.18	-0.08	-0.05	**-0.34**	0.02	-0.09	0.00	**-0.38**	0.11
DOSPERT Social Scale (Risk Taking)	-0.01	0.02	-0.01	0.08	0.14	0.00	-0.01	0.14	-0.01	-0.02	0.01	**0.73**
DOSPERT Social Scale (Expected Benefits)	0.00	-0.02	0.02	0.16	0.08	0.16	-0.05	0.27	-0.01	0.03	-0.02	**0.41**
DOSPERT Financial Scale (Risk Taking)	0.02	-0.02	0.05	-0.21	0.25	0.16	-0.06	0.00	0.12	-0.07	0.03	**0.34**
DKEFS Color Word Interference: Inhibition vs. Combined Naming + Reading Scaled (task)	0.02	-0.02	-0.06	-0.04	0.02	0.03	-0.02	-0.07	-0.03	0.06	0.06	0.13

Bold font denotes salient loading coefficients (>|.30|). We indicated if measures were derived from tasks, as well as the assessed time periods for all self-report measures that were not assessing traits.

Abbreviations:
ANT: Attention Network Test; ASRM: Adult Self Report; ATQ: Adult Temperament Questionnaire; BDI: Becks Depression Inventory; CFQ: Cognitive Failures Questionnaire; DKEFS: Delis-Kaplan Executive Functioning System; DOSPERT: Domain Specific Risk Taking Scale; NEO: Neo Five Factor Inventory; Penn CNP: Penn Computerized Neurocognitive Battery; STAI: State Trait Anxiety Inventory; UPPS: UPPS Impulsive Behavior Scale, WASI VCI: Wechsler Abbreviated Scale Intelligence Verbal Comprehension Index; WASI PRI: Wechsler Abbreviated Scale Intelligence Perceptual Reasoning Index; WIAT: Wechsler Individual Achievement Test

Supplementary Table 6: Factor Phi Correlations

	Internalizing	General Psychiatric	Effortful Control	Executive Function	Sensation Seeking	Unethical Behavior	Urgency	Openness/ Sensitivity	Extraversion/Sociability	Risk Perception	Negative Affect (Trait)	Social Risk-Taking
Internalizing	1.00	0.43	0.37	-0.03	-0.11	0.06	0.26	0.20	-0.32	0.07	0.36	-0.06
General Psychiatric	0.43	1.00	0.25	-0.01	0.05	0.16	0.23	0.19	-0.09	0.04	0.23	0.06
Effortful Control	0.37	0.25	1.00	0.06	0.05	0.17	0.34	0.08	-0.19	-0.07	0.20	0.01
Executive Function	-0.03	-0.01	0.06	1.00	0.09	0.13	-0.12	0.19	-0.05	-0.18	0.07	0.16
Sensation Seeking	-0.11	0.05	0.05	0.09	1.00	0.27	0.15	0.16	0.10	-0.20	-0.13	0.17
Unethical Behavior	0.06	0.16	0.17	0.13	0.27	1.00	0.26	0.00	-0.06	-0.27	0.05	0.19
Urgency	0.26	0.23	0.34	-0.12	0.15	0.26	1.00	0.04	0.05	-0.05	0.21	0.06
Openness/Sensitivity	0.20	0.19	0.08	0.19	0.16	0.00	0.04	1.00	0.06	0.04	0.15	0.23
Extraversion/ Sociability	-0.32	-0.09	-0.19	-0.05	0.10	-0.06	0.05	0.06	1.00	0.09	-0.12	0.07
Risk Perception	0.07	0.04	-0.07	-0.18	-0.20	-0.27	-0.05	0.04	0.09	1.00	0.06	-0.17
Negative Affect (Trait)	0.36	0.23	0.20	0.07	-0.13	0.05	0.21	0.15	-0.12	0.06	1.00	-0.02
Social Risk-Taking	-0.06	0.06	0.01	0.16	0.17	0.19	0.06	0.23	0.07	-0.17	-0.02	1.00

Supplementary Table 7a: Demographics & Diagnoses by Subtype

Subtype	Reward Type	Cognitive Type	Relief Type	Controls	Reward to Cognitive Type	Reward to Relief Type	Cognitive to Relief Type
Number of Participants	69	70	34	420	-	-	-
Age (Years)	37.7 (13.2)	**42.2 (12.1) ***	41.3 (14.2)	38.0 (13.3)	**P < .05 ***	ns	ns
Gender: M/F	**37/32 *****	27/43	14/20	115/305	ns	ns	ns
Education -Years Completed	**14.8 (2.0) ***	**14.4 (2.3) *****	15.1 (2.0)	15.4 (2.1)	ns	ns	ns
Past Alcohol UD (*N*) [M,F]	31 [11,20]	28 [7, 21]	16 [6,10]	0	ns	ns	ns
Past Cannabis UD (*N*) [M,F]	16 [9,7]	9 [3,6]	5 [3,2]	0	ns	ns	ns
Past Multi/Other UD (*N*) [M,F]	22 [17,5]	33 [17,16]	13 [5,8]	0	ns	ns	ns
Current Smoker (N)	**15 *****	**16 *****	2	28	ns	**P < .05 ***	**P < .05 ***
Tobacco Use (including smokeless): Times Using/Day	**3.8 (7.6) *****	**3.9 (18.2) ****	1.2 (4.3)	0.9 (3.3)	ns	ns	ns
Race (White/African-American/Other)	**41/19/9 ***	46/17/7	**10/21/3 *****	316/68/36	ns	**P < .01 ****	**P < .001 *****
Past 6 months Days Drunk (# Days)	**4.9 (7.6) ***	2.5 (5.4)	**5.9 (14.4) ***	2.5 (7.4)	**P < .05 ***	ns	ns
Past 6 months Nonmedicinal Substance Use (# Days)	**15.9 (35.4) *****	1.7 (4.3)	**9.1 (31.1) ***	2.7 (15.4)	**P < .01 ****	ns	ns
# Past Internalizing Disorders (%)	0.36 (0.71)	0.30 (0.49)	**0.50 (0.62) ***	0.25 (0.53)	ns	ns	ns
Anxiety (N)	0	0	0	1	-	-	-
Phobia (N)	2	1	0	4	-	-	-
Panic Disorder (N)	2	1	2	10	-	-	-
Obsessive Compulsive Disorder (N)	0	0	1	4	-	-	-
Depression or Dysthymic Disorder (N)	16	18	11	72	-	-	-
Eating Disorder or Body Dysmorphia (N)	1	2	1	4	-	-	-
Posttraumatic Stress Disorder (N)	3	0	0	9	-	-	-
# Past Externalizing Disorders (%)	0.02 (0.12)	0.03 (0.17)	**0.12 (0.36) *****	0.02 (0.14)	ns	**P < .01 ****	**P < .05 ***
Attention-deficit/hyperactivity disorder (N)	3	2	4	6	-	-	-
Bipolar Disorder (N)	0	0	2	0	-	-	-
Other Psychosis (eg, Schizophrenia) (N)	0	0	0	0	-	-	-

# Current Internalizing Disorders (%)	0.07 (0.26)	0.11 (0.32)	**0.79 (0.95) *** **	0.15 (0.47)		**P < .001 *** **	**P < .001 *** **
Anxiety (N)	3	4	8	15	-	-	-
Phobia (N)	1	3	2	14	-	-	-
Panic Disorder (N)	0	0	3	3	-	-	-
Obsessive Compulsive Disorder (N)	0	0	3	4	-	-	-
Depression or Dysthymic Disorder (N)	0	0	12	20	-	-	-
Eating Disorder or Body Dysmorphia (N)	0	1	2	4	-	-	-
Posttraumatic Stress Disorder (N)	1	0	5	6	-	-	-
# Current Externalizing Disorders (%)	0.04 (0.21)	0.01 (0.12)	0.09 (0.29)	0.03 (0.17)	ns	ns	ns
Attention-deficit/hyperactivity disorder (N)	3	1	3	11	-	-	-
Bipolar Disorder (N)	0	0	1	0	-	-	-
Other Psychosis (eg, Schizophrenia) (N)	0	0	0	2	-	-	-

UD = Use Disorder, CAARS = Conners' Adult ADHD Rating Scales

Age, Edu, Tobacco, Days Drunk, Nonmed Substance Use, & Comorbidity Columns: Mean (SD)

Bold font denotes Subtype differs from Controls; p<.05, p<.01**, p<.001*** (first three columns)*

Supplementary Table 7b: Clinical Characteristics by Subtypes							
	Reward Type	Cognitive Type	Relief Type	Controls	Reward Type to Cognitive Type	Reward Type to Relief Type	Cognitive Type to Relief Type
Number of Participants	69	70	34	420	-	-	-
ASR: Anxious/Depressed (past 6 months)	52.7 (3.8)	**51.7 (3.0) ***	**67.6 (7.9) *****	53.5 (6.8)	ns	**P < .001 *****	**P < .001 *****
ASR: Withdrawn (past 6 months)	**51.8 (3.4) ***	52.7 (3.9)	**63.1 (8.0) *****	53.4 (6.3)	ns	**P < .001 *****	**P < .001 *****
ASR: Somatic Complaints (past 6 months)	53.5 (4.4)	53.9 (4.5)	**61.4 (7.6) *****	53.5 (6.0)	ns	**P < .001 *****	**P < .001 *****
ASR: Thought Problems (past 6 months)	52.5 (4.2)	50.8 (1.4)	**58.6 (7.3) *****	52.0 (5.0)	**P < .01 ****	**P < .001 *****	**P < .001 *****
ASR: Attention Problems (past 6 months)	54.6 (4.8)	52.9 (3.9)	**63.4 (9.9) *****	54.1 (6.7)	**P < .05 ***	**P < .001 *****	**P < .001 *****
ASR: Aggressive Behavior (past 6 months)	52.6 (3.5)	52.0 (3.2)	**60.6 (6.8) *****	52.6 (5.3)	ns	**P < .001 *****	**P < .001 *****
ASR: Rule-Breaking Behavior (past 6 months)	**56.2 (5.9) *****	52.7 (4.2)	**61.2 (7.6) *****	53.1 (5.7)	**P < .001 *****	**P < .001 *****	**P < .001 *****
ASR: Intrusive (past 6 months)	53.5 (4.9)	51.4 (2.5)	**55.1 (5.0) ****	52.4 (4.9)	**P < .01 ****	ns	**P < .001 *****
Beck Depression Inventory Total (past 2 weeks)	5.7 (5.6)	4.5 (3.8)	**18.9 (7.0) *****	5.5 (6.7)	ns	**P < .001 *****	**P < .001 *****
Trauma Symptom Checklist (past 2 months)	20.1 (12.0)	18.8 (9.4)	**45.3 (14.5) *****	18.5 (13.3)	ns	**P < .001 *****	**P < .001 *****
CAARS: Inattention/Memory Problems	48.0 (9.0)	45.1 (7.5)	**55.3 (11.8) *****	47.2 (8.9)	**P < .05 ***	**P < .001 *****	**P < .001 *****
CAARS: Hyperactivity/Restlessness	**48.8 (8.4) ****	45.1 (5.7)	**55.1 (10.5) *****	45.8 (7.6)	**P < .01 ****	**P < .01 ****	**P < .001 *****
CAARS: Impulsivity/Emotional Liability	46.1 (6.8)	**47.0 (6.4) ***	**54.7 (8.5) *****	45.0 (7.0)	ns	**P < .001 *****	**P < .001 *****
CAARS: Problems with Self-Concept	45.9 (7.4)	46.6 (7.8)	**60.3 (10.2) *****	46.1 (8.8)	ns	**P < .001 *****	**P < .001 *****
CAARS: ADHD Index	46.4 (8.1)	45.2 (7.6)	**58.8 (9.2) *****	45.0 (8.1)	ns	**P < .001 *****	**P < .001 *****
CAARS: Inattention/Memory Problems	48.0 (9.0)	45.1 (7.5)	**55.3 (11.8) *****	47.2 (8.9)	**P < .05 ***	**P < .001 *****	**P < .001 *****
ASR = Adult Self Report, CAARS = Conners' Adult ADHD Rating Scales							
Independent Samples t-test was used to compare each Subtype to one another and to Controls.							
Mean (SD); Bold font denotes Subtype differs from Controls; p<.05, p<.01**, p<.001*** (first three columns)*							

Supplementary Table 8: Latent Factor Score Comparison Between Subtypes

Factor	Reward Type	Cognitive Type	Relief Type	Reward Type to Cognitive Type	Reward Type to Relief Type	Cognitive Type to Relief Type
Number of Participants	69	70	34	-	**-**	**-**
Internalizing	-0.163 (0.68)	-0.116 (0.62)	1.706 (0.69)	ns	**P < .01 ****	**P < .01 ****
General Psychiatric	-0.042 (0.49)	-0.307 (0.37)	1.595 (0.96)	**P < .01 ****	**P < .01 ****	**P < .01 ****
Effortful Control	0.158 (0.99)	-0.102 (0.81)	0.598 (1.31)			**P < .01 ****
Executive Function	0.113 (0.97)	-0.225 (0.88)	-0.217 (0.81)	**P < .05 ***	ns	ns
Sensation Seeking	0.695 (0.79)	-0.504 (0.61)	0.001 (1.04)	**P < .01 ****	**P < .01 ****	**P < .05 ***
Unethical Behavior	0.443 (0.98)	-0.322 (0.63)	0.288 (1.01)	**P < .01 ****	ns	**P < .01 ****
Urgency	0.316 (0.81)	-0.180 (0.73)	-0.542 (1.18)	**P < .01 ****	**P < .01 ****	ns
Openness/Sensitivity	0.407 (0.96)	-0.419 (0.67)	0.632 (0.89)	**P < .01 ****	ns	**P < .01 ****
Extraversion/Sociability	0.270 (0.97)	0.015 (0.72)	0.668 (1.01)	ns	ns	**P < .01 ****
Risk Perception	-0.462 (1.14)	0.198 (0.70)	0.362 (0.87)	**P < .01 ****	**P < .01 ****	ns
Negative Affect (Trait)	-0.211 (0.78)	-0.214 (0.80)	0.904 (0.69)	ns	**P < .01 ****	**P < .01 ****
Social Risk-Taking	0.599 (0.95)	-0.153 (0.67)	-0.135 (0.91)	**P < .01 ****	**P < .01 ****	ns

Mean (SD). All comparisons Holm-Bonferroni corrected. Bold font denotes significant comparisons; p<.05, p<.01**, p<.001***.*

Supplementary Table 9: Effect Sizes (Cohen's d) for Average Factor Scores by Subtype			
Factor	Reward Type to Cognitive Type	Reward Type to Relief Type	Cognitive Type to Relief Type
Internalizing	0.07	**2.66***	**2.80***
General Psychiatric	**0.61***	**2.20***	**2.60***
Effortful Control	0.30	0.38	**0.64***
Executive Function	**0.36***	0.37	0.01
Sensation Seeking	**1.70***	**0.75***	**0.59***
Unethical Behavior	**0.93***	0.16	**0.72***
Urgency	**0.64***	**0.85***	0.36
Openness/Sensitivity	**1.00***	0.24	**1.30***
Extraversion/Sociability	0.30	0.40	**0.75***
Risk Perception	**0.70***	**0.81***	0.21
Negative Affect (Trait)	0.003	**1.50***	**1.50***
Social Risk-Taking	**0.91***	**0.80***	0.02
Asterisk and boldface denote comparison of factor scores by subtype that were significant following Holm-Bonferroni correction.			

Supplementary Table 10: Comparing consistency of results for subtype to controls with results between subtypes

Significant Factors (to Controls)	Reward Type to Controls	Reward Type to Cognitive Type	Reward Type to Relief Type
Sensation Seeking	Yes	Yes	Yes
Social Risk-Taking	Yes	Yes	Yes
Unethical Behavior	Yes	Yes	No
Openness/Sensitivity	Yes	Yes	No
Risk Perception	Yes	Yes	Yes
Extraversion/Sociability	Yes	No	Yes
Urgency	Yes	Yes	Yes

Significant Factors (to Controls)	Cognitive Type to Controls	Cognitive Type to Reward Type	Cognitive Type to Relief Type
Sensation Seeking	Yes	Yes	Yes
General Psychiatric	Yes	Yes	Yes
Openness/Sensitivity	Yes	Yes	Yes
Unethical Behavior	Yes	Yes	Yes
Executive Function	Yes	Yes	No
Negative Affect (trait)	Yes	No	Yes
Risk Perception	Yes	Yes	No
Urgency	Yes	Yes	No
Extraversion/Sociability	Yes	No	Yes

Significant Factors (to Controls)	Relief Type to Controls	Relief Type to Reward Type	Relief Type to Cognitive Type
Internalizing	Yes	Yes	Yes
General Psychiatric	Yes	Yes	Yes
Negative Affect	Yes	Yes	Yes
Extraversion/Sociability	Yes	No	Yes
Openness/Sensitivity	Yes	No	Yes
Effortful Control	Yes	No	Yes

Supplementary Table 11: Predicting on Past 6 Months Days Binge-Drinking

	Resting-state Network (Laird et al., 2011)	Region (Glasser et al., 2016)	Area (Glasser et al., 2016)	Graph Theory measure	Increase or Decrease	p-FDR
**Reward Type**	Motor Planning	7. Mid Cingulate	R_6ma	Local Efficiency	Decrease	0.0051
	Motor Planning	8. Premotor	L_6d	Betweenness	Increase	0.0003330600344
	Motor Planning	7. Mid Cingulate	R_6ma (44)	Participation Coeff	Decrease	0.0346
	Motor Planning	8. Premotor	R_6d (54)	Participation Coeff	Decrease	0.0151
	DorsalFrontoparietal	7. Mid Cingulate	L_7AL	Local Efficiency	Decrease	8.26E-05
**Cognitive Type**	**No results**	-	-	-	-	-
**Relief Type**	Higher Visual	15. TPOJ	L_STV (208)	Participation Coeff.	Decrease	0.0052
	Higher Visual	14. Lateral Temporal	L_PHT (317)	Participation Coeff.	Decrease	0.0478
	Salience	19. Medial prefrontal	L_a24	Betweenness	Increase	0.0053
	Salience	19. Medial prefrontal	L_p24	Betweenness	Increase	0.0209
	VentralFrontoparietal	21. Inferior frontal	L_44	Betweenness	Increase	0.0209
	Value/Reward	21. Inferior frontal	L_p47r	Betweenness	Increase	0.0053
	Auditory	15. TPOJ	R_TPOJ1 (139)	Participation Coeff.	Decrease	0.0048
	Motor Planning	8. Premotor	L_6d (234)	Participation Coeff.	Decrease	0.0478
